# The critical role of plasma membrane H^+^-ATPase activity in cephalosporin C biosynthesis of *Acremonium chrysogenum*

**DOI:** 10.1371/journal.pone.0238452

**Published:** 2020-08-31

**Authors:** Alexander Zhgun, Mariya Dumina, Ayrat Valiakhmetov, Mikhail Eldarov

**Affiliations:** 1 Research Center of Biotechnology RAS, Moscow, Russia; 2 Skryabin Institute of Biophysics and Physiology of Microorganisms, RAS, Pushchino, Russia; University of California Riverside, UNITED STATES

## Abstract

The filamentous fungus *Acremonium chrysogenum* is the main industrial producer of cephalosporin C (CPC), one of the major precursors for manufacturing of cephalosporin antibiotics. The plasma membrane H^+^-ATPase (PMA) plays a key role in numerous fungal physiological processes. Previously we observed a decrease of PMA activity in *A*. *chrysogenum* overproducing strain RNCM 408D (HY) as compared to the level the wild-type strain *A*. *chrysogenum* ATCC 11550. Here we report the relationship between PMA activity and CPC biosynthesis in *A*. *chrysogenum* strains. The elevation of PMA activity in HY strain through overexpression of *PMA1* from *Saccharomyces cerevisiae*, under the control of the constitutive gpdA promoter from *Aspergillus nidulans*, results in a 1.2 to 10-fold decrease in CPC production, shift in beta-lactam intermediates content, and is accompanied by the decrease in *cef* genes expression in the fermentation process; the characteristic colony morphology on agar media is also changed. The level of PMA activity in *A*. *chrysogenum* HY *OE*::*PMA1* strains has been increased by 50–100%, up to the level observed in WT strain, and was interrelated with ATP consumption; the more PMA activity is elevated, the more ATP level is depleted. The reduced PMA activity in *A*. *chrysogenum* HY strain may be one of the selected events during classical strain improvement, aimed at elevating the ATP content available for CPC production.

## Introduction

Cephalosporins are a class of beta-lactam antibiotics with potent bactericidal action, low toxicity, and wide therapeutic range [1]. Numerous derivatives represent chemical modifications of the parent molecule cephalosporin C (CPC) produced by filamentous fungi *A*. *chrysogenum* [2]. During recent decades, significant progress has been made in the development of high-yielding (HY) CPC strains of *A*. *chrysogenum* after classical strain improvement (CSI) programs, as well as in the determination of CPC biochemical pathway, identification of the genes, responsible for beta-lactams biosynthesis, transport and transcriptional regulation [3]. These so-called *cef* genes are organized in two clusters on *A*. *chrysogenum* chromosomes and differ in the temporal expression patterns in the course of antibiotic biosynthesis. The “early” cluster (chromosome VI) contains genes for the first steps in CPC biosynthesis, *pcbAB* (encodes δ-(L-α-aminoadipyl)-L-cysteinyl-D-valine synthetase for ACV tripeptide production), *pcbC* (encodes isopenicillin N synthase for convention of ACV tripeptide to isopenicillin N; IPN), *cefD1* (encodes isopenicillin N-CoA synthetase), and *cefD2* (encodes isopenicillin N epimerase) for sequential conversion IPN to penicillin N (PenN). The “early” cluster also contains genes for proteins involved in pathway-specific transcriptional regulation (*cefR*) or transport of CPC biosynthesis intermediates between subcellular compartments (*cefM*, *cefP*) and out of the fungal cell (*cefT*). The “late cluster” (chromosome I) encodes two genes for the final steps of CPC biosynthesis, *cefEF* (encodes deacetoxycephalosporin C synthetase/ hydroxylase) and *cefG* (encodes deacetylcephalosporin-C acetyltransferase), responsible for three enzymatic activities with the sequential conversion of PenN to deacetoxycephalosporin C (DAOC), then deacetylcephalosporin C (**DAC**) and finally to CPC [4,5]. A number of different factors and processes which can also significantly affect CPC biosynthesis in *A*. *chrysogenum* were investigated, such as reactive oxygen species [6], autophagy [7–9], sulfur biosynthesis and endogenous S-adenosylmethionine content [10,11], transcription factors [12–15], etc. These achievements, together with the development of methods for genetic manipulation and “omics” technologies applied to *A*. *chrysogenum* [16,17], opened new opportunities for improvement of industrial strains [18,19].

Several strategies of genetic manipulation can be used to enhance the level of the target secondary metabolites (SM) production or modify the important strains parameters such as productivity, product export, the concentration of by-products, stress-strain characteristics, morphology and so on [19,20]. Targets for such manipulation are transcription factors, transmembrane transporters, proteins that regulate secretion, signal transduction pathways, cell surface receptors, and enzymes of primary and secondary metabolism [21]. The biosynthesis of CPC in *A*. *chrysogenum* is a complicated process involving enzymatic reactions, complex regulation and specific membrane transport steps. It was shown that transporters involved in the translocation of biosynthetic intermediates between subcellular compartments, as some specific reactions compartmentalized within the fungal cell, are essential for CPC production. They significantly contribute to the overall level of CPC production, affecting the ratio of the target product and its intermediates in the course of fermentation [22].

*A*. *chrysogenum cef* genes for beta-lactam transporters are localized in the “early” biosynthetic cluster [23,24]. It was assumed that CPC export to the culture medium is directed by CefT belonging to the major facilitator superfamily (MFS) antiporters [25], however, this observation was questioned in further studies [26]. The MFS transporters use the electrochemical gradient generated by the plasma membrane H^+^-ATPase (PMA) [27]. PMAs are major regulators of cytoplasmic pH and plasma membrane (PM) potential in eukaryotic cells, they generate proton motive force from ATP hydrolysis for the driving a lot of crucial transport processes, including nutrient uptake and export of secondary metabolites (SM) [28].

Previously we demonstrated the decreased PMA activity in *A*. *chrysogenum* HY strain [29,30]. This strain typically produces 9–12 grams of CPC during laboratory fermentation in shake flasks, 200–300 times higher than WT strains [29]. We have previously shown that the overproduction phenotype correlates with upregulation of *cef* genes [31], chromosomal rearrangements [5], as well as alterations in polyamine metabolism [32], in cell wall structure [33], in size of filamentous hyphae and conidia formation [34], in colony size and coloration [26], and has other physiological changes. The PMA1 defect may be one of the reasons for reduced strain growth rate and overall fitness, diminished resistance to abiotic stress and proficiency in nutrients [30]. So, the question arises as to whether such deficiency in PMA activity can affect the transport of target antibiotics, namely lowering of CPC export and/or decreasing nutrients uptake from the culture medium, thus influencing the strain growth rate. Here we report the results of correction this deficiency through the introduction of the plasma membrane H^+^-ATPase from *Saccharomyces cerevisiae* (PMA1_sc_) into *A*. *chrysogenum* HY strain. The choice of *S*. *cerevisiae PMA1* gene as a target for this replacement was motivated by extensive studies of biochemistry, genetics, physiology, trafficking, and assembly of PMA1_sc_[35]. It was shown that its fusions with fluorescent proteins retain functionality [36] and heterologous PMA1 proteins are functional in yeast cells [37]. The introduction of exogenous *PMA1* gene into *A*. *chrysogenum* cells also gave the possibility to independently monitor the expression of endogenous and foreign genes and to easily distinguish the relative contribution of endogenous and exogenous genes for PMA activity of recombinant strains.

## Materials and methods

### Strains of microorganisms

*A*. *chrysogenum* ATCC 11550 (WT, wild type Brotzu isolate, [38]) and *A*. *chrysogenum* RNCM 408D (HY, high yielding CPC producer, derived from the WT, [29]) were used for functional analysis, comparative gene expression and genetic transformation procedures. *E*.*coli* XL1-blue was used for plasmids construction. *S*. *cerevisiae* SY4, *S*. *cerevisiae* YPH857 [39]–were used as recipients for the study of membrane topology and functional properties of the PMA1-TagYFP fusion protein. *Agrobacterium tumefaciens* AGL0 was used a donor for transferring the PMA1-TagYFP expression cassette into *A*. *chrysogenum* RNCM 408D. The genotypes of the strains used are given in Table 1.

**Table 1:** Strains of microorganisms used in the study.

Strain	Genotype	Source
*Acremonium chrysogenum* ATCC 11550 (WT)	Wild type, cephalosporin C producer	ATCC, USA, [38]
*A*. *chrysogenum* RNCM 408D (HY)	High yielding cephalosporin C producer, *ATCC 11550*:: *CSI program*	Laboratory collection, [29]
*Agrobacterium tumefaciens* AGL0	*EHA101 pTiBo542ΔT-region Mop*^+^	[40]
*Escherichia coli* XL1-Blue	*recA1 endA1 gyrA96 thi-1 hsdR17 supE44 relA1 lac [F’proAB lacIqZΔM15 (Tetr)]*	“Stratagene”, USA
*Saccharomyces cerevisiae* S288C	*Matα SUC2 gal2 mal mel flo1 flo8-1 hap1 ho bio1 bio6*	[41]
*S*. *cerevisiae* YPH857	*MATα*, *ura3-52*, *lys2-801*, *ade2-101*, *trp1Δ63*, *his3Δ200*, *leu2Δ1*, *cyh2R*	[42]
*S*. *cerevisiae* SY4	*MAT*a, *ura3–52*, *leu2–3*,*112*, *his4–619*, *sec6–4ts GAL2*, *pma1*::*YIpGAL-PMA1*	[43]
*S*. *cerevisiae* SY4/ pZEN36-H	*MAT*a, *ura3–52*, *leu2–3*,*112*, *his4–619*, *sec6–4ts GAL2*, *pma1*::*YIpGAL-PMA1*, pZEN36-Н	This study
*S*. *cerevisiae* YPH857/ pZEN36b	*MATα*, *ura3-52*, *lys2-801*, *ade2-101*, *trp1Δ63*, *his3Δ200*, *leu2Δ1*, *cyh2R*, pZEN36b	This study
AcPS2	RNCM 408D *hyg res*::*pTrpC-hph-t35S*::*pGpdA-PMA1sc-taqYFP-tPGK* (pZEN36c, TFO event 2)	This study
AcPS4	RNCM 408D *hyg res*::*pTrpC-hph-t35S*::*pGpdA-PMA1sc-taqYFP-tPGK* (pZEN36c, TFO event 4)	This study
AcPS6	RNCM 408D *hyg res*::*pTrpC-hph-t35S*::*pGpdA-PMA1sc-taqYFP-tPGK* (pZEN36c, TFO event 6)	This study
AcPS10	RNCM 408D *hyg res*::*pTrpC-hph-t35S*::*pGpdA-PMA1sc-taqYFP-tPGK* (pZEN36c, TFO event 10)	This study
AcPS11	RNCM 408D *hyg res*::*pTrpC-hph-t35S*::*pGpdA-PMA1sc-taqYFP-tPGK* (pZEN36c, TFO event 11)	This study
AcPS20	RNCM 408D *hyg res*::*pTrpC-hph-t35S*::*pGpdA-PMA1sc-taqYFP-tPGK* (pZEN36c, TFO event 20)	This study
AcCefT6	RNCM 408D *hyg res*::*pTrpC-hph-t35S*::*pGpdA-cefT-taqCFP-tPGK* (pZEN33, TFO event 6)	[26]

### Plasmids construction

For the expression of PMA1-TagYFP fusion protein in *S*. *cerevisiae* two plasmids were obtained, starting from YCp2HSE-PMA1 vector [43] with *PMA1* from *S*. *cerevisiae* X2180 [44,45]. PMA1 coding sequence was amplified from YCp2HSE-PMA1 using primers PMA_H3_F/ PMA_Age-R (Table 2), the fragment was treated with *Hind*III/ *Age*I restriction enzymes and cloned into the *Hind*III/ *Age*I treated vector pTaqYFP-N (“Evrogen”, Russia). The resulting pZEN17 intermediate construct encodes PMA1-TagYFP C-terminal fusion with a 13 amino acids linker sequence GAGAGAGAGPVAT. The pZEN36-H vector for the expression PMA1-TagYFP in *S*. *cerevisiae* under the control of thermos-inducible *2HSE* promoter was obtained by cloning of the 2053 bp KpnI/ SspI fragment from pZEN17 (with 3`-terminal coding sequence of *Pma1-tagYFP*) into *Kpn*I/ *Ecl236*II cut vector YCp2HSE-PMA1. The pZEN36b vector for constitutive PMA1-TagYFP expression was obtained by replacement of 2*HSE* promoter with the *TEF1* promoter from *Ashbya gossypii*. For this end the 415 bp PCR fragment obtained on the template of pZEM5 plasmid [34] with primers Tef1_Xho_up/ Tef1_ Hind_dw (Table 2) was cloned into XhoI/ *Hind*III cut vector pZEN36-H. For heterologous expression of PMA1-TagYFP in *A*. *chrysogenum* the binary pZEN36c vector was constructed. For this purpose the fragment containing the PMA1-TagYFP and SV40 polyA sequences was obtained from pZEN36b as a *Spe*I-“blunt”/ *Hind*III 4036 bp DNA fragment and inserted into the *Pme*I/ *Hind*III site of pZEN33 [26]. The description of used plasmids is given in Table 3.

**Table 2:** Oligonucleotides used in this study.

Oligonucleotide	Sequence (5’ → 3’)	Purpose	Source
PMA_H3_F	GTAAGCTTAATGACTGATACATCATCCTCTTCATCA	fusion of *PMA1*_*sc*_ and *tagYFP* sequences in pZEN17	pYCp2HSE-PMA1, [43,45]; pTagYFP-N
PMA_Age_R	GACCGGTCCGGCACCAGCACCCGCCCCTGCTCCGGTTTCCTTTTCGTGTTGAGTAGAG		
Tef1_Xho_up	CTGCTCGAGGATCCCCGGGTTAATTAAGGCGC	introducing of *TEF1* promotor from *A*. *gossypii* (instead of *2HSE*) for *PMA1*_*sc*_ expression in pZEN36	pZEM5, [34]
Tef1_Hind_dw	CAGAAGCTTGTTGTTTATGTTCGGATGTGA		
Pma1ac_F	ATGGCTGACAACAAGGCTGCCGGC	isolation of *pma1* from cDNA of *A*. *chrysogenum* ATCC 11550 and RNCM 408D	Genbank JPKY01000044.1
Pma1ac_R	TTGCGACTTCTCGTGCTGTGTGGA		
PMAac_seq01	GGTATCACCCAGAACCGTGG	sequencing of *pma1*, isolated from cDNA of *A*. *chrysogenum* ATCC 11550 and RNCM 408D	
PMAac_seq02	TTCTCGCCACCGACGTACAG		
PMAac_seq03	ACGGAGTACTTGTGCTGGGG		
PMAac_seq04	GAACTCGAGGACCTTGTACT		
PMAac_seq05	ACCAGCAGACTGGGAGACGA		
PMAac_seq06	CCGCAAATGACACCAAAATC		
PMAac_seq07	CACACGTTGTGGATAGAAAA		
Npt3F	CGATATCCTCCCTGATCGACCGGACGCAGA	PCR screening of *A*. *chrysogenum* transformants for absence of pZEN36c (for *npt3* gene of aminoglycoside phosphotransferase for kanamycin resistance)	pZEN16, [34,46]
Npt3R	GCCGATGTGGATTGCGAAAACTGGGAAGAA		
Vir1	GGCTACATCGAAGATCGTATGAATG	PCR screening of *A*. *chrysogenum* transformants for absence of *A*. *tumefaciens* (for *vir*-genes)	Genbank AB027257.1; [34]
Vir2	GACTATAGCGATGGTTACGATGTTGAC		
Hyg1	GTTACATGTAGATCTGATATTGAAGGAGCATTTTTTGGGCTTGGC	PCR screening of *A*. *chrysogenum* transformants for *hygB*	pZEN16, [46]
Hyg2	CTGATTAATACTAGTTAACTGGTTCCCGGTCGGCATCTAC		
GKR1	GCAACATCCTGGGCCACAAG	PCR screening of *A*. *chrysogenum* transformants for *PMA1*_*sc*_*-taqYFP*	pTaqYFP-N; [26]
GKF1	CGGTACAGCTCGTCCATGCCGT		
actq1	CCGGTTTCGCCGGTGATGATGCT	qPCR of *act* for *A*. *chrysogenum* gamma-actin, the major component of the cytoskeleton	Genbank JN836733; [26]
actq2	TGCTCAATGGGGTAGCGCAG		
pcbABq3	AGGCATCGTCAGGTTGGCCG	qPCR of *pcbAB* for *A*. *chrysogenum* L- δ-(L-α-aminoadipyl)-L-cysteinyl-D-valine synthetase	Genbank E05192, [31]
pcbABq4	CCGGAGGGGCCATACCACAT		
pcbCq1	CTAGGTCGCGACGAGGACTTCT	qPCR of *pcbC* for *A*. *chrysogenum* isopenicillin N synthase	Genbank M33522, [31]
pcbCq2	CACGTCGGACTGGTACAACACC		
cefD1q1	GTTGGTGAGCGGCTTCGGGATA	qPCR of *cefD1* for *A*. *chrysogenum* isopenicillin N-CoA synthetase	Genbank AJ507632, [31]
cefD1q2	AGTAGGTGCGGTCACCGTTGGG		
cefEFq3	CTTCTACTTGACCGAGAGCGGCC	qPCR of *cefEF* for *A*. *chrysogenum* deacetoxycephalosporin C synthetase/ hydroxylase	Genbank AJ404737, [31]
cefEFq4	TAGTCCGAGTACTTGCCCGTCTC		
cefGq3	CTCCTGGAGCCATATGGAAGCGC	qPCR of *cefG* for *A*. *chrysogenum* deacetylcephalosporin-C acetyltransferase	Genbank M91649, [31]
cefGq4	GGTGCGCAGCTTGGTTCGAGAC		
cefRq1	GGGACTGGAGTTTTGCTGCGGA	qPCR of *cefR* for *A*. *chrysogenum* regulator of *CPC* biosynthesis	Genbank HM230824, [31]
cefRq2	GGACGACCGAGGTACAGAGACCACA		
cefTq3	TGTTGTCGGATTCGGTGTCGG	qPCR of *cefT* for *A*. *chrysogenum* MSF transporter	Genbank AJ487683; [26]
cefTq4	TTCCACATATCGGCAAGGGTGC		
cefMq1	TTTATCCAGGAGGAGCGCGGTC	qPCR of *cefM* for *A*. *chrysogenum* MSF transporter of penicillin N	Genbank AM231815, [31]
cefMq2	TGTCGTAGGCGGTTCACCTTGC		
cefPq1	AATGCGACCCCGAGGAGTACGT	qPCR of *cefP* for *A*. *chrysogenum* MSF transporter of isopenicillin N	Genbank AM231816, [31]
cefPq2	CCATCCCAGGAATGTTGTCGGC		
PMAac_q1	GGACATGCTCCAGACCGACC	qPCR of *pma1* for *A*. *chrysogenum* plasma membrane H^+^-ATPase	Genbank JPKY01000044.1
PMAac_q2	CACCAAAATCGACCCAGTCCTC		
PMAsc_q1	CAGCTGTCGTTACCACCACTATGGCCGTC	qPCR of *PMA1*_*sc*_ for *S*. *cerevisiae* plasma membrane H^+^-ATPase	pYCp2HSE-PMA1, [43]
PMAsc_q2	GCCAAACAAGCAGTCAACATCAAGTCGTCTGGAG		
PMAsc_q3	AGAAGTGTCGAAGACTTCATGGCTGCTA	PCR screening of *A*. *chrysogenum* transformants for *PMA1*_*sc*_*-taqYFP* and qPCR *PMA1*_*sc*_*-taqYFP*	pZEN36
PMAsc_q4	ACGCTGAACTTGTGGCCGTGCACGTC		

**Table 3:** Plasmids used in this study.

Plasmid	Description	Source
pTagYFP-N	Source of *tagYFP* cording sequence	“Evrogen”, Russia
pYCp2HSE-PMA1	Yeast centromere plasmid for *PMA1* expression under the control of thermoinducible promoter *2HSE*	[43]
pZEM5	The binary vector for *apl*I expression in fungi cells under the control of *TEF1* promotor from *A*. *gossypii*	[34]
pZEN17	Vector for *PMA1* and *tagYFP* fusion	This study
pZEN33	The binary vector for *cefT* from *A*. *chrysogenum* expression under the control of gpdA promotor from *A*. *nidulans* in fungi cells	[26]
pZEN36-H	Yeast centromere plasmid for with *2HSE*-*pma1*-*tagYFP*-*Sv40polyA* expression cassette	This study
pZEN36b	Yeast centromere plasmid for with P*TEF1*_ag_*-pma1-tagYFP-Sv40polyA* expression cassette	This study
pZEN36c	The binary vector for *PMA1*_*sc*_ transferring and expression under the control of gpdA promotor from *A*. *nidulans* in fungi cells	This study

### The identification of *A*. *chrysogenum Pma1*

Total RNA was extracted from *A*. *chrysogenum* WT and HY strains after 120 h of fermentation, as described previously [31], mRNA fraction was obtained with oligo (dT)_30_ magnetic particles (“Sileks”, Russia), cDNA was obtained by M-MLV reverse transcriptase with oligo (dT)_15_ primers kit (“Sileks”) according to recommendation of manufacturer. *PMA1* was amplified from cDNA with primers PMAac_F/ PMAac_R and sequenced with primers PMAac_seq01 –PMAac_seq07 (Table 2). The nucleotide sequence of *PMA1* from *A*. *chrysogenum* HY is available from GenBank under accession number MK641804.1; the corresponding amino acid sequence accession number–QDF45217.1. Sequences of fungal plasma membrane ATPases were aligned using VectorNTI software *v*.8.0 [47]. Genbank accession numbers for analyzed sequences are provided in Table 4.

**Table 4:** Fungal plasma membrane H^+^-ATPases.

Fungi, H^+^-ATPase	Amino acids	% Similarity	% Identity	GenBank accession no.
*A*. *chrysogenum* ATCC 11550, PMA1	929	100	100	KFH44673.1, [16]
*A*. *chrysogenum* ВКМ F4081D, PMA1	929	100	100	QDF45217.1, This study
*Neurospora crassa*, PMA1	920	91,6	85,0	J02602, [48]
*S*. *cerevisiae* X2180, PMA1	918	83,7	74,5	X03534.1, [45]
*Schizosaccharomyces pombe*, PMA1	919	83,0	71,8	J03498, [49]
*Candida albicans*, PMA1	895	82,0	73,3	M74075, [50]
*Aspergillus fumigatus*, PMA1	988	60,0	45,0	AY040608, [51]
*Aspergillus nidulans*, PMAA	990	59,7	44,9	AF036763, [52]

### Genetic transformations of fungal cells

The *S*. *cerevisiae* SY-4 and YPH857 strains were transformed using lithium acetate method [53]. Transformation of *A*. *chrysogenum* cells was performed by *Agrobacterium tumefaciens*-mediated transformation (ATMT) [54]. The electroporation of *A*. *tumefaciens* AGL0 with pZEN36c binary vector, cocultivation of *A*. *chrysogenum* RNCM 408D with *A*. *tumefaciens* AGL0/ pZEN36c cells, transferring on Hybond N membrane (“GE Healthcare”, USA) and selection of transformants on hygromycin B- supplemented agar were done as described before [26,34].

### Analysis of *A*. *chrysogenum* transformants

Hygromycin-resistant *A*. *chrysogenum* clones obtained by ATMT procedure were subjected for PCR-screening to verify the presence of the expression cassette (with pairs of primers PMAsc_q3/ GKF1, or PMAsc_q3/ PMAsc_q4, or Hyg1/ Hyg2, or GKR1_N/ GKF1_N); absence of agrobacterial contamination (primers Vir1 /Vir2) and absence of pZEN36c vector contamination (primers Npt3F/ Npt3R –to amplify the sequence, corresponding to non-transferring part of binary vector) (Table 2). Selected “positive” clones were analyzed by Southern blot hybridization. Genomic DNA isolated according to the protocol [55], treated with the *Asi*A1, separated in 1% agarose and transferred to the Amersham Hybond-XL membrane (“GE Healthcare”, USA) under alkaline transfer conditions. The DNA fragment with *PMA1*_*sc*_-*tagYFP* sequence was obtained after PCR of pZEN36c with primers GKR1_N/ GKF1_N, labeled with DecaLabel DNA Labeling Kit ("Fermentas", Lithuania) and used in hybridization procedure. Visualization was performed with Typhoon Trio^+^ Imager (“GE Healthcare”, USA), as described previously [5].

### Culture media and growth conditions *for A*. *chrysogenum*

Preparation of *A*. *chrysogenum* seed cultures and fermentation of the selected strain in the defined production media were carried out using the media and conditions described previously [31]. Samples were taken at the following time points 0 (start of fermentation), 48 and 120 h of fermentation and further used for fluorescence microscopy, HPLC analysis, proteomic analysis, determination of intracellular ATP content, plasma membrane H^+^-ATPase activity and isolation of total RNA.

### Fluorescence microscopy of *S*. *cerevisiae* and *A*. *chrysogenum* cells

Micrographs of *A*. *chrysogenum* RNCM 408D/ PMA1-TagYFP cells were obtained using an Olympus BX2 microscope (“Olympus”, Japan) with the set of fluorescent filters UMNIBA3 (excitation 470–495 nm; dichroic mirror 505 nm, emission 510–550 nm).

### HPLC analysis of beta-lactams

Concentration of CPC and beta-lactam biosynthesis intermediates in the culture broth were determined in the CTAB/ acetonitrile/ orthophosphoric acid/ water mobile phase on a YMC-Pack ODS-A chromatographic column (“YMC CO.”, Japan) with a particle diameter of 5 μm at a flow rate of 1.0 ml/ min of the mobile phase, detection 254 nm.

### Measurements of intracellular ATP levels and H^+^-ATPase activity

ATP extraction from *A*. *chrysogenum* cells and ATP quantification was performed using luciferin-luciferase ATP bioluminescence assay kit («Merck», USA) and LKB 1250 Luminometer («LKB», Sweden) as described in [56]. H^+^-ATPase activity in PM preparations of *A*. *chrysogenum* was measured as previously described in the presence and in the absence of 100 μM sodium orthovanadate, a specific inhibitor of H^+^-ATPase activity of PM [30,57]. 100 mM deoxyglucose was added to the test samples as a negative control of the source of carbon during the preincubation of cells [57].

### RNA extraction, cDNA preparation and qPCR analysis

Isolation of total RNA from *A*. *chrysogenum* cells after different stages of fermentation, cDNA synthesis, qPCR reactions, data processing and normalization was performed as described previously [26,31]. Primer sequences used to evaluate expression levels of *pma1* and *cef* genes are given in Table 2.

## Results

### Identification of the gene encoding the plasma membrane H^+^-ATPase in *A*. *chrysogenum*

To reveal the phenomenon of the decreased PMA1 activity in *A*. *chrysogenum* HY strain [30] we identified the gene encoding main plasma membrane H^+^-ATPase *(AcPma1)* in this organism. Based on predicted gene encoding the PMA1-like protein in *A*. *chrysogenum* ATCC 11550 (GenBank: JPKY01000044.1, region 69388–72840, [16]) we amplified from cDNA, isolated from WT and HY strains, the full-length copies corresponding to the sequences of spliced mRNA. The cDNA sequencing showed the correct joining of all three predicted exons (GenBank: JPKY01000044.1 join complement: 69388..69434, 69490..71824, 72433..72840, [16]). The *AcPma1* cDNA sequence from WT strain was 100% identical to the CDS predicted from the annotated genomic sequence; the *AcPma1* sequence from HY strain (GenBank: MK641804.1) had a single silent mismatch T1740C, that does not change 555Gly. As a result, *AcPma1* genes from WT and HY strains encode identical proteins (GenBank: KFH44673.1 and QDF45217.1, respectively). We also demonstrated that these sequences encode the main plasma membrane H^+^-ATPase in *A*. *chrysogenum*, after performing proteomic analysis by tandem mass spectrometry for *A*. *chrysogenum* WT and HY strains. Description of the proteomic analysis for *A*. *chrysogenum* WT and HY strains is provided in S1 File; the proteomic data for the *A*. *chrysogenum* WT strain are given in S1 Table, the proteomic data for *A*. *chrysogenum* HY strain are given in S2 Table. For both strains, the protein products with molecular weight of 101363 that completely corresponded to the GenBank sequences: KFH44673.1 and QDF45217.1, respectively, were found. The genome of *A*. *chrysogenum* ATCC11550 is predicted to encode another PMA-like protein (Genebank: KFH43902.1). Our proteomic analysis did not reveal the presence of peptides derived from this protein. Thus, KFH43902.1 is a probable orthologue of *S*. *cerevisiae PMA2* gene, encoding a minor nonessential plasma membrane H^+^-ATPase, highly homologous to *PMA1*, but expressed at a very low level and only during the haploid cycle or under stress conditions [58]. The alignment of AcPMA1 amino acid sequence with fungal plasma membrane H^+^-ATPases revealed the highest level of homology with model PMA1 enzymes from *Neurospora crassa* (85% of identity, 91,6% similarity) and *S*. *cerevisiae* (74.5% of identity, 83% similarity). The identity above 70% was observed with PMA1 from *S*. *pombe* and *C*. *albicans*, while *Aspergillus* PMA1 orthologues showed the lowest levels of homology (Table 4).

### *AcPma1* expression in *A*. *chrysogenum* WT and HY strains

**Fig 1:**
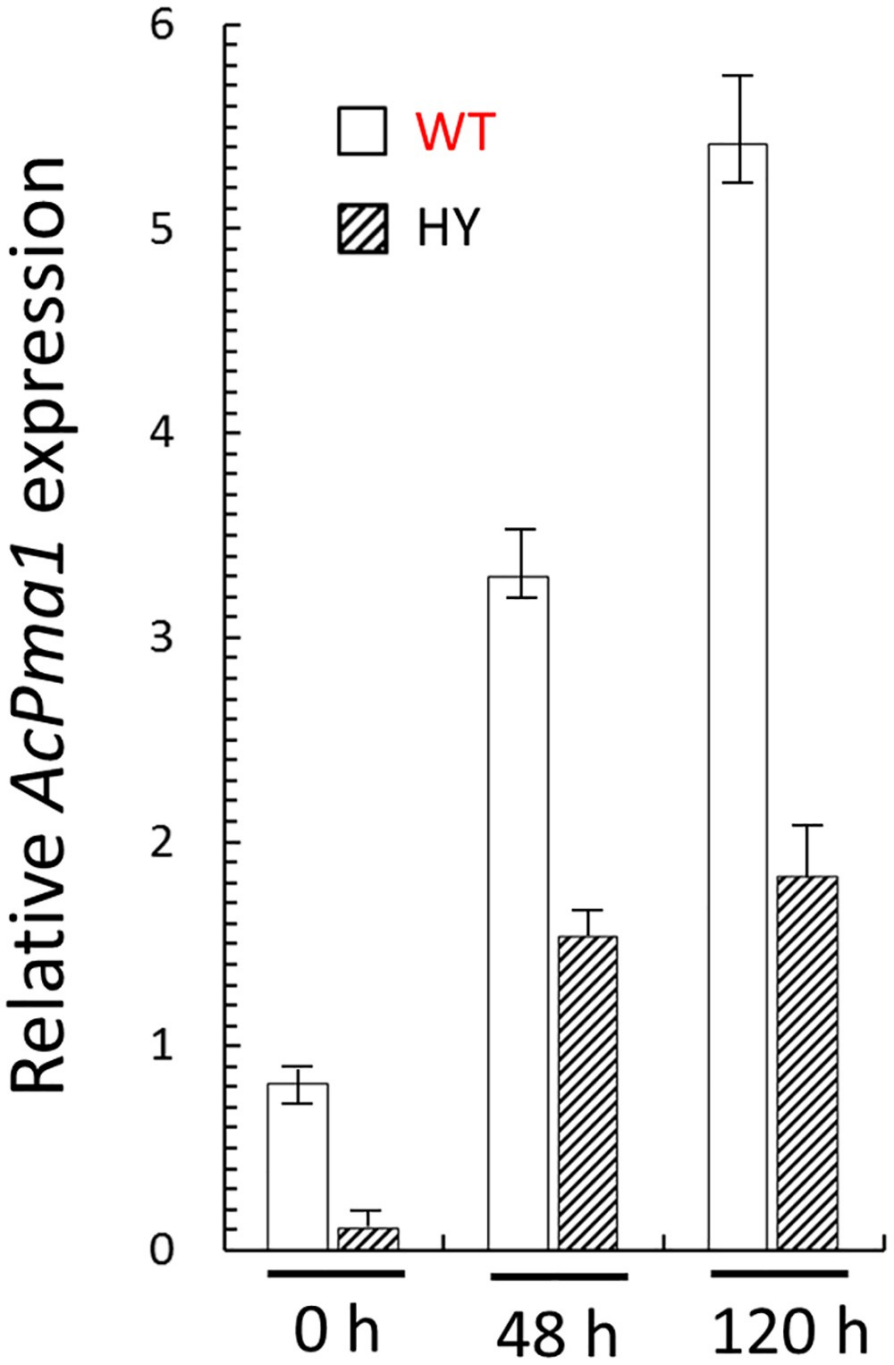
Expression dynamics of *AcPma1* gene in *A*. *chrysogenum* WT and HY strains. 0, 48 and 120 h of fermentation. Data are means ± SD, n = 3.

Since the primary sequence of PMA1 is unchanged in the HY strain, the decrease in H^+^-ATPase activity [30] should be due to trans-acting factors. To establish a possible change in regulation at the transcription level, *pma1* expression was studied during the fermentation of *A*. *chrysogenum* WT and HY strains (Fig 1). It turned out that in both strains, there was an increase in the *pma1* expression throughout the entire fermentation period. At the same time, RNA levels were lower for HY at each analyzed point. At the beginning of fermentation, downregulation was 10 times or more, then, the difference decreased, but remained significant, 2–5 times. The detected downregulation of *pma1* in the HY strain may be the principal factor for decreasing the PMA activity in HY strain.

### *A*. *chrysogenum* HY/PMA1_sc_-TaqYFP transformants

To study the possible relationship between the H^+^-ATPase activity of the plasma membrane and CPC biosynthesis in *A*. *chrysogenum*, the PMA1 from *S*. *cerevisiae*, the most studied fungal P type H^+^-ATPase, was used as a genetic engineering tool. This enzyme has been comprehensively characterized by numerous studies [43,45]. Earlier, we have shown that our variant of PMA1_sc_-TaqYFP fusion protein with a long flexible spacer is correctly targeted to the plasma membrane in *S*. *cerevisiae* cells and efficiently couples with CefT, MFS transporter of beta-lactams from *A*. *chrysogenum* [26]. In the current study, we measured the PMA activity in S. cerevisiae/ PMA1_sc_-TaqYFP strains under the control of constitutive (YPH857/pZEN36b) and heat-inducible (SY4/pZEN36-H) promotors (S3 Table). The PMA activity in YPH857 recombinant clones with constitutive expression of *PMA1-TaqYFP* under the control of TEF1 promoter from *A*. *gossypii* was increased 1.3–1.5 fold (S3 Table), which could be related to simultaneous expression with the chromosomal *PMA1* copy. The PMA activity in SY4/pZEN36-H strain (under glucose inactivation of the chromosomal copy of *PMA1* and heat-shock activation of *2HSE-PMA1-TaqYFP*) was very close to PMA activity in recipient SY4 strain (S3 Table). That means the PMA1 C-end fusion through GAGAGAGAGPVAT linker with TaqYFP did not influence the PMA activity and may be used as a genetic engineering tool. This is important, as it has been previously shown that PMA1-GFP fusion has a 3 fold reduced PMA activity [57]. The possibility of efficient heterologous expression of *Pma1* from filamentous fungi in *S*. *cerevisiae* cells was previously demonstrated [59]; however, in our experiments, we did not obtain an efficient expression for *AcPma1* in *S*. *cerevisiae* cells.

**Fig 2:**
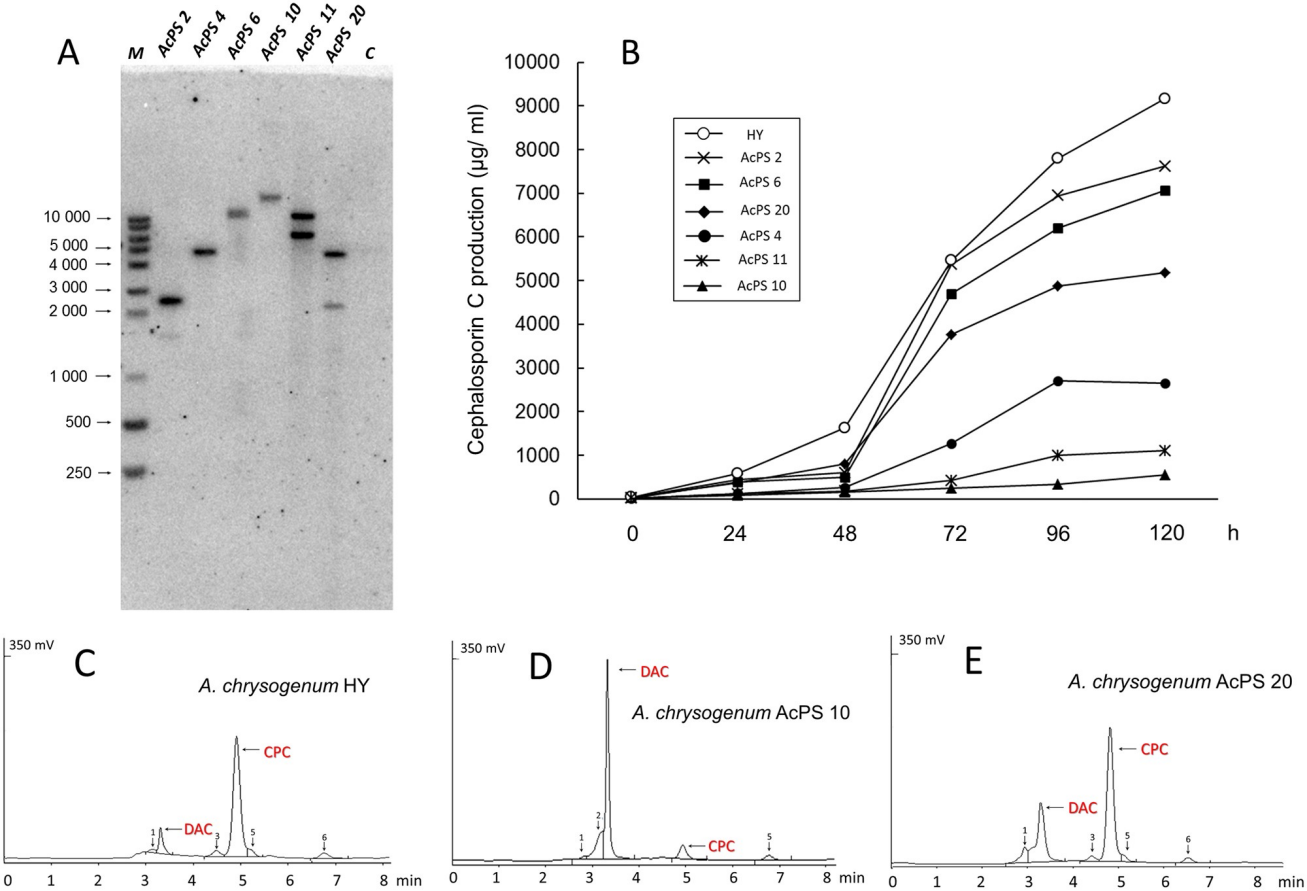
Analysis of *A*. *chrysogenum* HY/ pZEN36c transformants. (A) Southern blot hybridization of *Age*I-digested genomic DNA *PMA1*_*sc*_*-tagYFP* specific probe: *M*–GeneRuler 1 kb DNA Ladder (Thermo Fisher Scientific, USA); *AcPS 2*, *4*, *6*, *10*, *11*, *20* –*Age*I-digested DNA of *A*. *chrysogenum* HY/ pZEN36c transformants; *C*–*Age*I- digested DNA of *A*. *chrysogenum* HY. (B) CPC production of *A*. *chrysogenum* HY and AcPS strains 2, 4, 6, 10, 11, 20 after cultivation on a seed medium (0 h) and fermentation medium (48, 120 h). (C–E) HPLC analysis of beta-lactam production after 120 h of fermentation *A*. *chrysogenum* strains: HY (C), AcPS10 (D) and AcPS20 (E).

For heterologous expression in *A*. *chrysogenum* cells we constructed pZEN36c vector with target *PMA1*_*sc*_*-tagYFP* gene under the control of gpdA promoter from *A*. *nidulans*, inserted into the T-DNA region of the binary vector pZEN16 [46], and *hygR* gene under the control TrpC promoter from *Aspergillus niger* for the selection of transformants. After the ATMT procedure, optimized for HY strain previously [26], the 126 HygB-resistant transformants were obtained, 36 transformants were further verified by PCR screening for the presence of target *PMA1*_*sc*_*-tagYFP* gene, absence of bacterial contamination with the donor *A*. *tumefaciens* strain and absence of non-transferring part of pZEN36c binary vector. Six selected transformants were also characterized by Southern blotting with the probe specific to *PMA1*_*sc*_*-tagYFP* gene (Fig 2A). All six transformants have different patterns and apparently arose due to independent transformation events; three among them carried a single copy of *PMA1*_*sc*_*-tagYFP* insertion (AcPS4, AcPS6, and AcPS10), while another 3 contained two copies of inserted expression cassette (AcPS2, AcPS11, and AcPS20).

**Fig 3:**
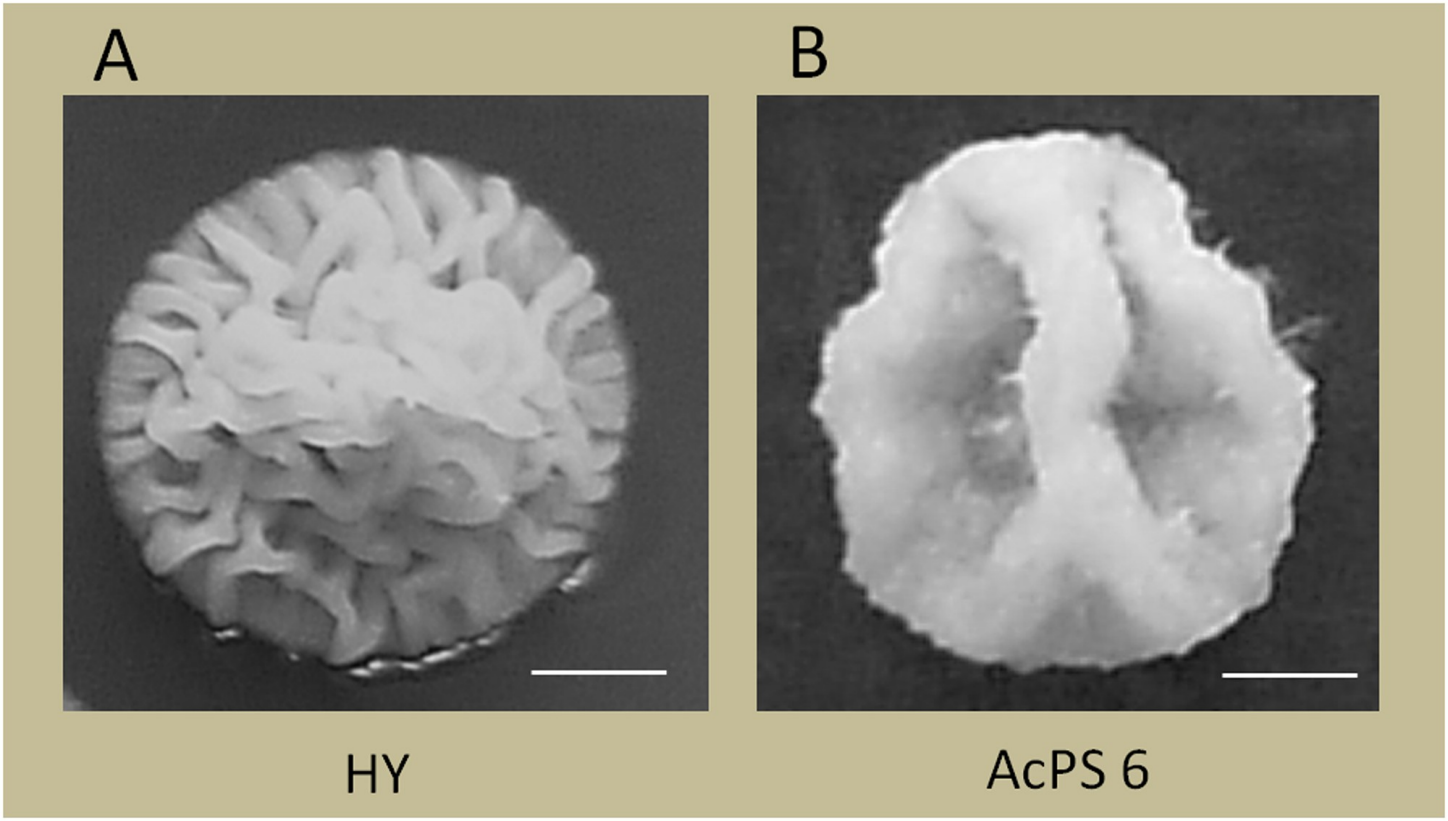
The morphology of *A*. *chrysogenum* colonies after 12 d cultivation on agar medium, 26°C. *A*. *chrysogenum* strains: HY (A), AcPS6 (B). Scale bar = 2 mm.

All selected transformants demonstrated characteristic morphological changes on agar medium (Fig 3). Transformed colonies showed a reduction of surface roughness and formed one major groove instead of many small and tortuous grooves that are typical for 1-week colonies of recipient strain HY; the colony size was not changed. Such phenotype did not depend on inserted copy numbers of *PMA1*_*sc*_ and was detected for all AcPS strains.

**Fig 4:**
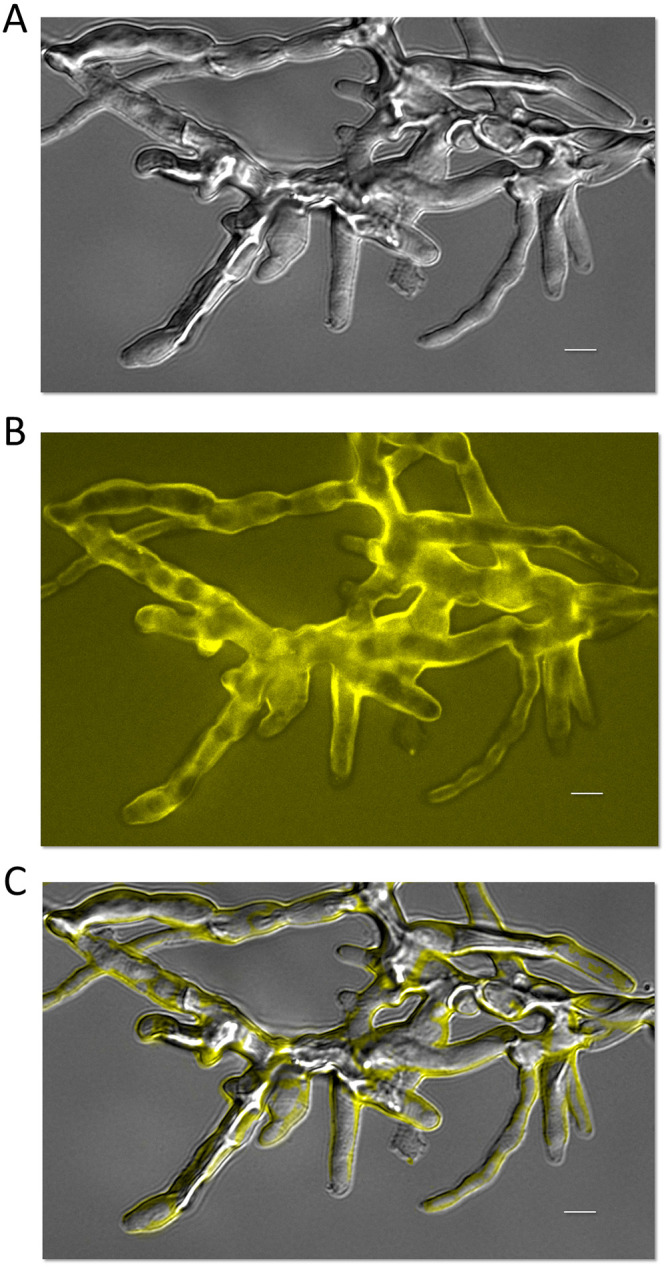
Fluorescent analysis of PMA1_sc_-TagYFP expression in *A*. *chrysogenum* AcPS6 strain. 48 h of fermentation, 24°C. (A) Photograph made in transmitted light. (B) Fluorescent analysis. (C) Superimposition of the cell structure in transmitted light (A) and fluorescence (B). Scale bar = 5 μm.

We also determined the subcellular localization of PMA1_sc_-TagYFP in selected transformants (Fig 4). The PMA1_sc_ fused with TagYFP correctly incorporated into plasma membrane of *A*. *chrysogenum* HY. Fluorescent microscopy of the hyphal cells for AcPS strains revealed PM specific fluorescence, similar to that detected in *N*. *crassa* cells, expressing PMA1 fused with GFP from C-terminal [60]. PMA1_sc_-TagYFP in *A*. *chrysogenum* also localized at the PM at distal regions of mycelium and in completely developed septa, but not at the tips, in apical regions (Fig 4B and 4C).

### PMA activity, CPC production and ATP content in *A*. *chrysogenum* strains

**Fig 5:**
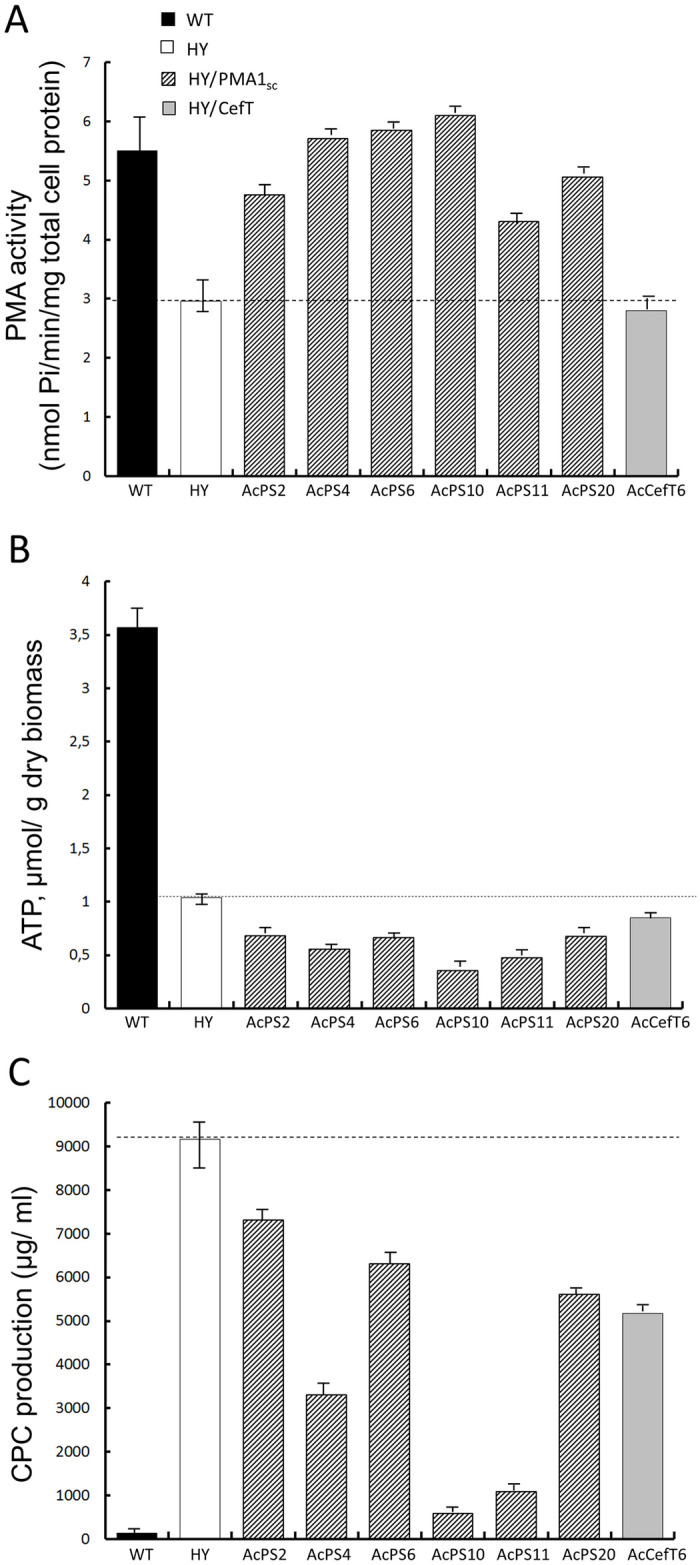
PMA activity, ATP content and CPC production of *A*. *chrysogenum* strains. 120 h of fermentation. (A) PMA activity. (B) ATP content. (C) CPC production. Dashed lines indicate levels corresponding to the HY strain.

We measured the PMA activity, CPC production, and ATP content in *A*. *chrysogenum* WT, HY, AcPS2, 4, 6, 10, 11, 20, and AcCefT6 strains (Fig 5). All *PMA1*_*sc*_-recombinants of HY strain demonstrated the increased PMA activity, up to its level in WT strain (Fig 5A). This elevation of PMA activity was accompanied with a significant decrease in the intracellular ATP content, 1.5–3 fold relative to HY strain-recipient, and 5–10 fold relative to WT strain (Fig 5B). Notably, all AcPS strains demonstrated the inverse ratio between PMA activity and ATP content; highest PMA1 activity in recombinants was accompanied by the most noticeable decrease in ATP content (Fig 5A and 5B).

To estimate, whether ATP content depletion in HY-recombinants is due to the elevation of PMA activity just a side-effect of the ATMT procedure, we measured the PMA activity and ATP content in previously obtained recombinant strain AcCefT6 from *A*. *chrysogenum* HY with expression cassette *cefT-TaqCFP*, inserted in the same binary vector [26,46]. The MFS beta-lactam transporter of *A*. *chrysogenum* CefT localizes into the same compartment as PMA1, fungal plasma membrane [26]. The PMA1 activity in AcCefT6 was very close to that in the recipient HY strain, and intracellular ATP content was only slightly reduced (Figs 3A and 5B).

CPC production in all AcPS strains was reduced from 1.2 to 12 fold as compared to HY strain (Figs 2B and 3C). The decrease in the production of the target metabolite was not correlated with the copy number. The reduction was accompanied by a simultaneous increase in the content of DAC, the immediate precursor of CPC. The typical HPLC analysis for HY strain reveals less than 10–15% of DAC content (Fig 2C), for AcPS10, more than 90% (Fig 2D), for AcPS20, 30–35% (Fig 2E).

**Fig 6:**
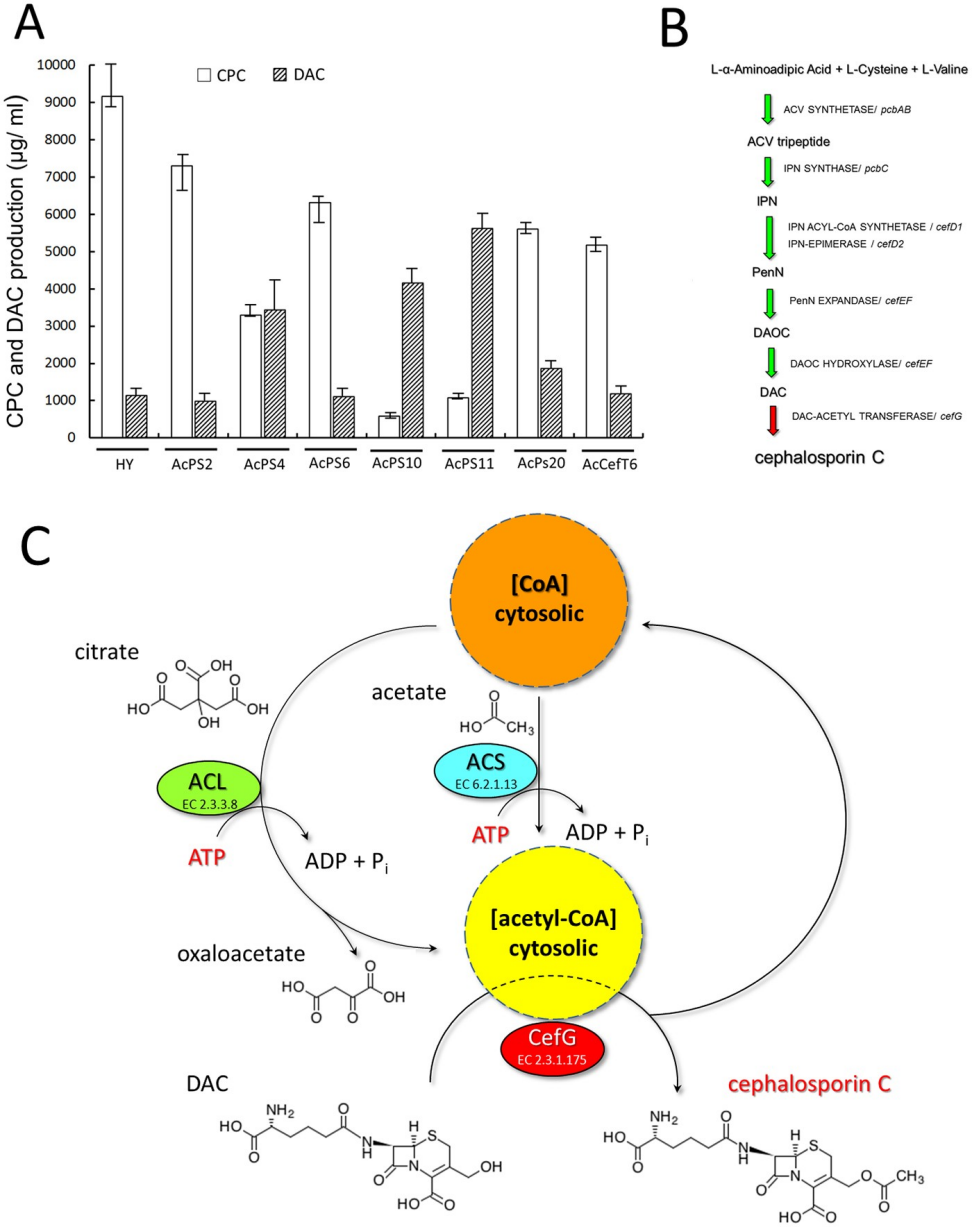
Analysis of CPC and DAC production in *A*. *chrysogenum*. (A) CPC and DAC production in *A*. *chrysogenum* HY, AcPS and AcCefT6 strains after 120 h of fermentation. (B) CPC biosynthetic pathway. (C) DAC to CPC conversion in *A*. *chrysogenum*. ACV tripeptide–δ-L-α-aminoadipyl-L-cysteinyl-D-valine tripeptide, IPN–isopenicillin N, PenN–penicillin N, DAOC–deacetoxycephalosporin C, DAC–deacetylcephalosporin C, CPC–cephalosporin C, ACL–ATP-citrate lyase, ACS–acetyl-CoA synthetase, CefG–deacetylcephalosporin-C acetyltransferase.

The overall balance of beta-lactam cephems (CPC and DAC) in HY and its recombinants is shown in Fig 6A. According to the ratio of cephems, all strains can be divided into three groups, with CPC/ DAC ratio of 80–90% (HY, AcPS2, AcPS6, AcPS20, and AcCefT6), equal CPC/ DAC production (AcPS4) and with CPC/ DAC ratio of 10–20% (AcPS10, AcPS11). The reduction of beta-lactam production in the AcCefT6 strain was previously discussed [26].

**Fig 7:**
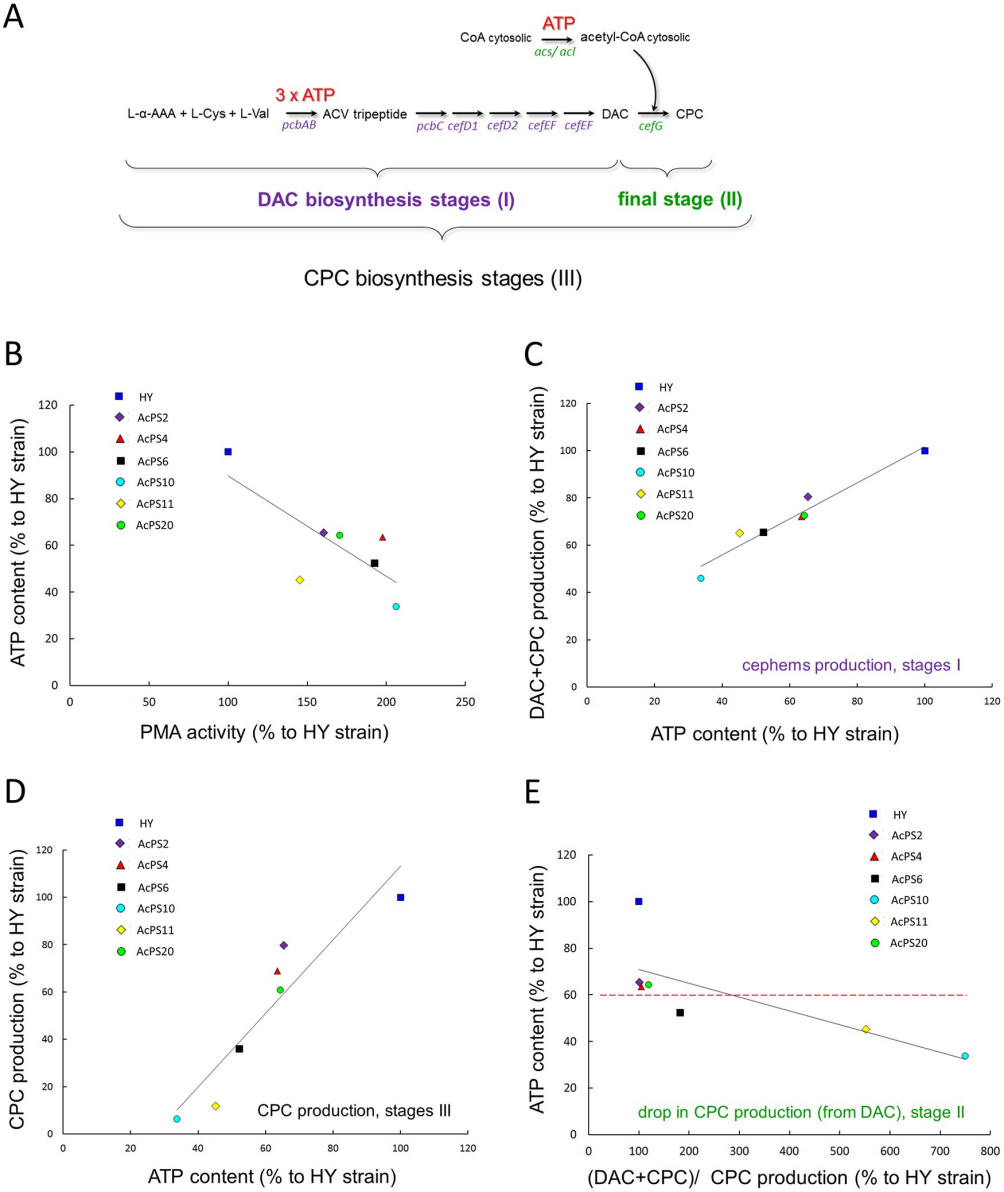
Analysis of ATP content for CPC production in *A*. *chrysogenum* strains after 120 h of fermentation. DAC–deacetylcephalosporin C, CPC–cephalosporin C. (A) ATP consuming on different stages of CPC production. (B) Ratio of ATP content (% to HY strain) to PMA activity (% to HY strain). (C) Ratio of DAC and CPC total production (% to HY strain) to ATP content (% to HY strain). (D) Ratio of CPC production (% to HY strain) to ATP content (% to HY strain). (E) Ratio of ATP content (% to HY strain) to (DAC + CPC)/ CPC (% to HY strain)–means DAC amount, unconverted to CPC (% to HY strain); the dashed line show the threshold of ATP content for effective DAC to CPC conversion.

The CPC biosynthesis is an ATP consuming process. At the first stage of this pathway the enzyme ACV synthetase utilizes 3 ATP molecules to sequentially activate the three amino-acid substrates to formaminoacyl-adenylates, in NRPS synthesis of ACV tripeptide (Fig 7A) [61]. But under unfavorable reaction conditions more than 20 mol of ATP are consumed per 1 mol of tripeptide formed. This increase has been attributed to the hydrolysis of intermediates, such as adenylates or amino acid thioesters [61]. The final stage of the CPC biosynthetic pathway is rate-limiting and estimated as a “bottleneck” for CPC biosynthesis [62] (Fig 6B). It is catalyzed by the enzyme CefG, uses DAC and acetyl coenzyme A (acetyl-CoA) as substrates, and occurs in the cytoplasm [4]. There are two potential sources of cytoplasmic acetyl-CoA in filamentous fungi: from citrate via ATP-citrate lyase (ACL; EC 2.3.3.8), which depends on citrate entering the cytoplasm from the mitochondrion, or from acetate via acetyl-CoA synthetase (ACS; EC 6.2.1.13) [63] (Fig 6C). In both reactions, one ATP molecule is consumed to produce one acetyl-CoA molecule (Figs 6C and 7A). The high levels of DAC are accumulated in many CPC-producing strains [64]. The total yield of CPC in industrial strains is limited, mainly, by the efficiency of the CefG-catalyzed reaction (EC 2.3.1.175) (Fig 6C). If this process is not effective, the DAC precursor is accumulated, and CPC yield falls. Various improved *A*. *chrysogenum* strains have a DAC/ CPC ration of 30–35% or more [62]. Since *cefG* overexpression in recombinant strains leads to the decreasing of DAC content and increasing in CPC yield, one of the limiting factors is inefficient *cefG* expression [62]. However, in *A*. *chrysogenum* HY strain, the amount of DAC did not exceed 10–15% of CPC yield [29]. This was achieved due to the random mutagenesis and selection for the decrease in DAC/ CPC ratio [29] and resulted in significant *cefG* upregulation [31]. Since two substrates (DAC and acetyl-CoA) are required for the CefG reaction, its efficiency is dependent on three factors: 1) DAC content, 2) acetyl-CoA content and 3) CefG amount (Fig 6C). Obviously, in improved *A*. *chrysogenum* HY strain, with effective DAC to CPC conversion, none of these factors limit the reaction. The depletion of ATP content in *A*. *chrysogenum* HY *OE*::*PMA1* strains (Fig 5B) leads to shift in CPC/ DAC ratio (Fig 6A) and has a downtrend with an increase in PMA activity (Fig 7B). The higher the PMA activity, the more the ATP content is depleted, the more CPC/ DAC ratio decreases (Figs 5 and 6A). Thus, in AcPS10 strain with the highest PMA1 activity, the ATP content is most severely depleted, and CPC/ DAC ratio in the most severely reduced (Figs 2D, 5 and 6A). Moreover, in all recombinants with significant depletion of the ATP content (AcPS4, AcPS10, and AcPS11) the main product of beta-lactams biosynthesis converts from CPC to DAC (Figs 5B and 6A). The total cephems (DAC + CPC) production depends on DAC biosynthetic stages (Fig 7A) and decreases with the depletion of ATP content (Fig 7C). The CPC production includes, in addition to DAC biosynthetic stages, one more ATP-consuming final stage (Fig 7A) and decreases more significantly with the depletion of ATP content (Fig 7D).

The depletion of the ATP content can influence the decrease in cytoplasmic acetyl-CoA content, by lowing the activity of ATP-consuming enzymes for cytoplasmic acetyl-CoA synthesis (ACL and ACS) (Fig 6C) and by shifting acetyl-CoA metabolism in mitochondria from acetyl-CoA biosynthesis to its oxidation for ATP synthesis [65]. That leads to the decrease in the DAC acetylation in CefG-catalyzed reaction and reduction in yield of target metabolite, CPC (Fig 6C). Obviously, there is a minimum threshold level of ATP content, after which the efficiency of the final stage rapidly falls (Fig 7E). For recombinants with 60–65% ATP content (AcPS2, AcPS4, and AcPS20 strains), DAC is converted to CPC at the level of HY strain. A drop in the ATP content to 50% leads to a 2-fold decrease in DAC to CPC conversion (Fig 7E, AcPS6). A further decrease in the ATP content leads to a sharp drop in reaction efficiency. The decrease to 45% leads to a 5.5-fold decrease in DAC to CPC conversion (AcPS11), the decrease in AcPS11 strain to 33% leads to a 7.5-fold decrease in DAC to CPC conversion (Fig 7E). It can be assumed that the threshold minimum of ATP content for efficient DAC to CPC conversion is very close to 60% from ATP content in *A*. *chrysogenum* HY strain (Fig 7E). The presence of a threshold concentration of ATP content for the CefG-catalyzed reaction explains such a large spread in the CPC production in HY/ PMA1 recombinants, the 1.2–10 fold (Fig 5C). The production of cephems drops by 45–80% and is in the trend with ATP content depletion, by 35–67% (Fig 7C). The reaction of DAC to CPC conversion has a threshold for ATP content depletion; the 35–40% of ATP content depletion does not influence CefG-catalyzed reaction, further depletion (up to 67%) has a downtrend of 2–7.5 folds decreasing in DAC to CPC conversion.

### Expression levels of homo- and heterologous *PMA1* and *cef* genes in *A chrysogenum* strains

**Fig 8:**
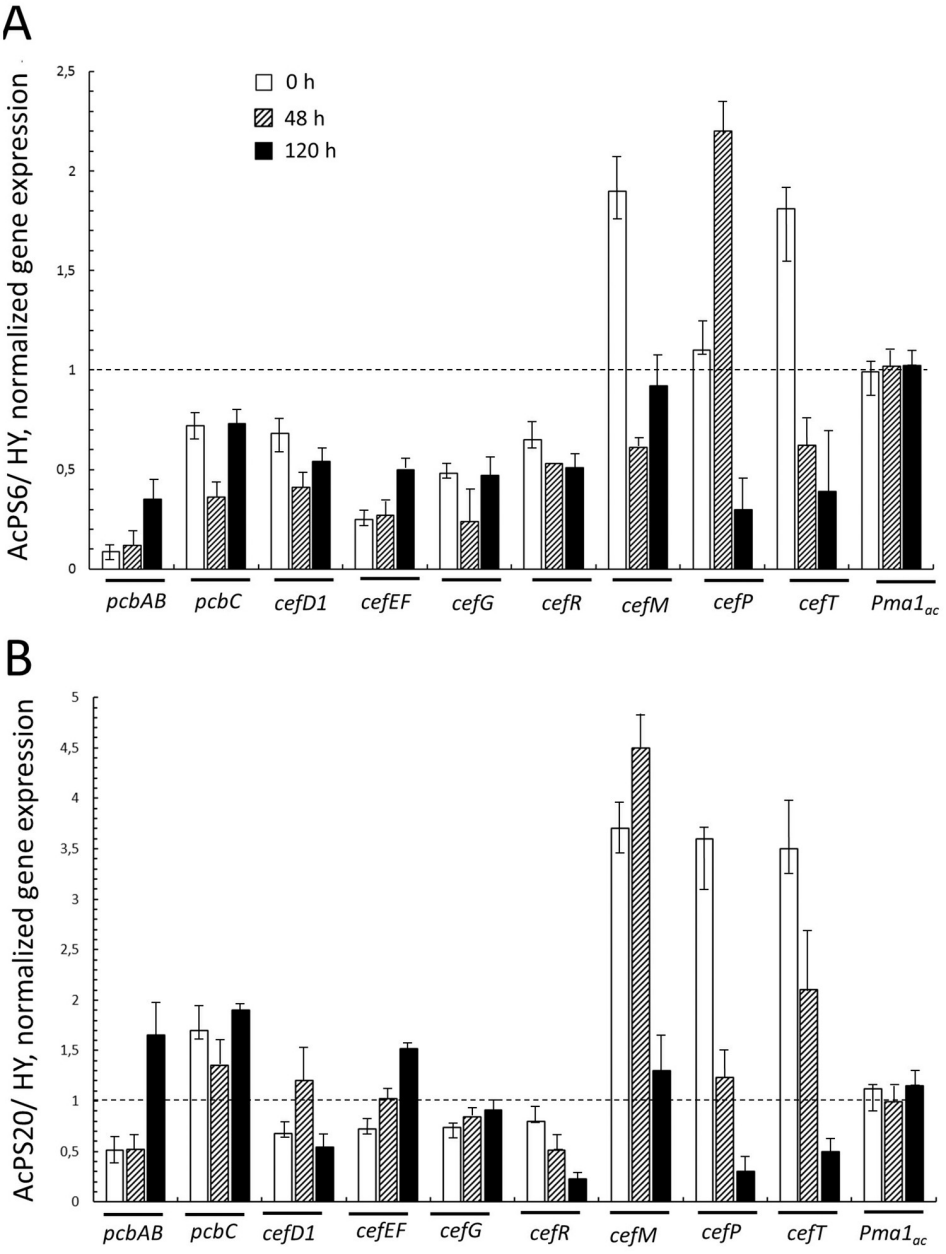
Relative expression of *cef* genes and *Pma1*_ac_ in *A*. *chrysogenum* HY/*PMA1*_*sc*_ strains. 0, 48, and 120 h fermentation of *A*. *chrysogenum*. (A) AcPS6/ HY. (B) AcPS20/ HY. The dashed lines show a comparative level of gene expression in the HY strain. Data are means ± SD, n = 3.

The analysis of the dynamics of *cef* genes expression for two chosen AcPS clones showed variable trends at studied fermentation timepoints (Fig 8). For the AcPS6 clone, all genes were downregulated 2–10 fold as compared to parent HY strain at the start, middle and end of fermentation period (0, 48 и 120 h). For the AcPS20 at all timepoints we observed upregulation of the *pcbC* (1.5–2.0 fold); downregulation of *cefG* and *cefR* (1.5–3 fold). Levels of *pcbAB*, *cefD1*, and *cefEF* mRNAs changed 1.5–2 folds in both directions and did not differ significantly from the levels observed for HY strain. The expression pattern of genes encoding MFS proteins with clearly different transport functions in the CPC pathway, such as translocation of the early intermediates between subcellular compartments and final antibiotic secretion from the cell, differed from that of the biosynthetic *cef* genes. *CefM*, *cefP*, and *cefT* were steadily upregulated with maximum expression levels observed at the start of fermentation.

**Fig 9:**
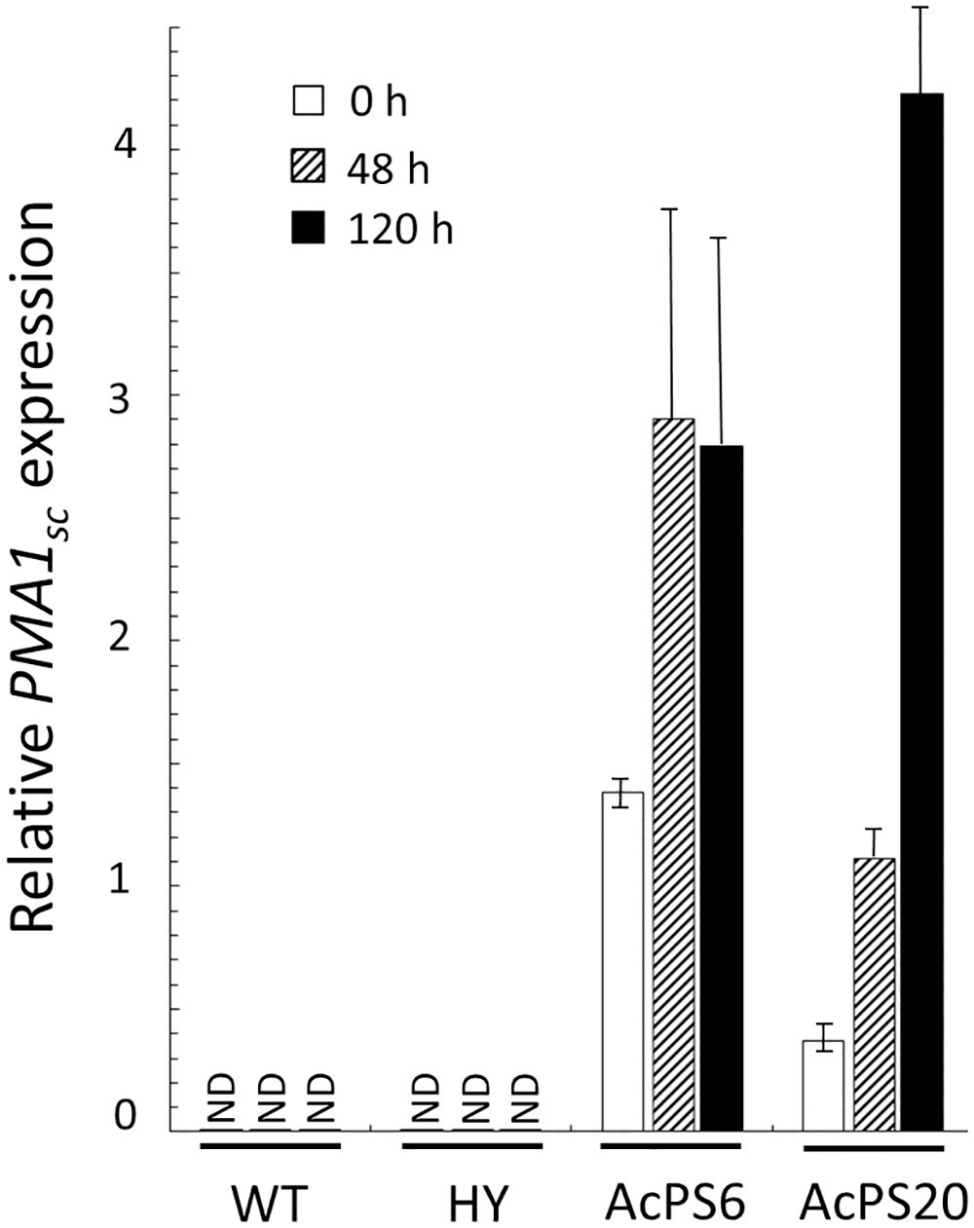
Expression dynamics of *PMA1*_*sc*_ gene in *A*. *chrysogenum* WT, HY, AcPS6 and AcPS20 strains. 0, 48 and 120 h of fermentation. Data are means ± SD, n = 3. ND, not detected.

The endogenous *AcPma1* gene expression levels were the same at the three timepoints (Fig 8). In contrast, expression of the heterologous *PMA1*_*sc*_*-TaqYFP* gradually increased towards the end of the fermentation period (Fig 9). A similar expression pattern was observed before in our recombinant *A*. *chrysogenum* clones, expressing the *cefT-taqCFP* fusion gene under the control of the same gpdA promoter and may reflect a specific pattern of regulation of this promoter in HY strain in fermentation conditions optimal for CPC production [26].

## Discussion

Industrial primary and secondary metabolite overproducing strains obtained through CSI programs often contain unwanted side mutations and “bottlenecks”, negatively affecting strain fitness, robustness, productivity, and adaptation to harsh fermentation conditions [66,67]. Whenever possible, these defects may be identified and corrected using modern “omics” techniques, systems biology and synthetic biology approaches, metabolic modeling, genome editing, reverse genetics etc [68,69]. Successful examples of the application of this strategy towards metabolic engineering of industrial beta-lactam producing strains include overexpression of *cefG* gene [62], introduction of a truncated gene copy for PacC transcription factor, modulation of strain morphology through manipulation with *Acatg1* [9] and *Acthi1* [10] genes, enhancing oxygen uptake by expression of bacterial hemoglobin gene [19].

P-type plasma membrane H^+^-ATPase plays an essential role in the physiology of fungal cells [28]. This proton pump generates the electrochemical proton-motive force across the membrane that drives the energy-dependent uptake of amino acids, sugars, nucleosides and inorganic ions [27], as well as the export of SM. In addition, H^+^ transport, mediated by this enzyme, contributes to the regulation of intracellular pH and surface pH along the hyphae [70]. The activity of beta-lactam transporters also depends on the transmembrane proton potential generated by proton translocating H^+^-PMA1 ATPase [71].

We demonstrated previously that CPC overproducing *A*. *chrysogenum* HY strain had reduced PMA activity [30]. The observed physiological changes in this strain are associated with generally reduced fitness, and stress-resistance, including marked growth rate reduction on solid and liquid medium [31,32] and may be due to the reduced PMA1 activity [72]. What is the molecular mechanism of this phenomenon? It could be caused by various factors such as direct inhibition of the enzyme, decrease in the amount of the enzyme, or by the several combinations of factors. In recent work, we showed that HY strain has increased intracellular content of polyamines (PAs) [32]. PAs can modulate ATPase pump activity, from inhibitory effects [73,74] to its activation [75]. In some organisms, different polyamines have the opposite effect. For instance, in pea roots, higher PA spermine inhibits H+-ATPase activity, whereas lower PA putrescine activates it [76]. In the HY strain, the putrescine content is extremely low (which is close to the putrescine content in WT strain), spermidine content is increased in 5.1 fold, the spermine content is increased in 4.5 fold [32]. Such a shift in PAs content could be the reason for decreasing PMA activity in CPC overproducing strain. From the other side, our proteomic analysis data shows that the total amount of PMA1 in HY strain is 45% lower than in the WT strain (S1 and S2 Tables). This data correlates with the downregulation of *AcPma1* in HY strain (Fig 1). The total decrease in PMA activity, measured in HY strain vs. WT strain (Fig 5A and S3 Table), may be associated simultaneously with reducing the total amount of the enzyme and its inhibition by PAs.

**Fig 10:**
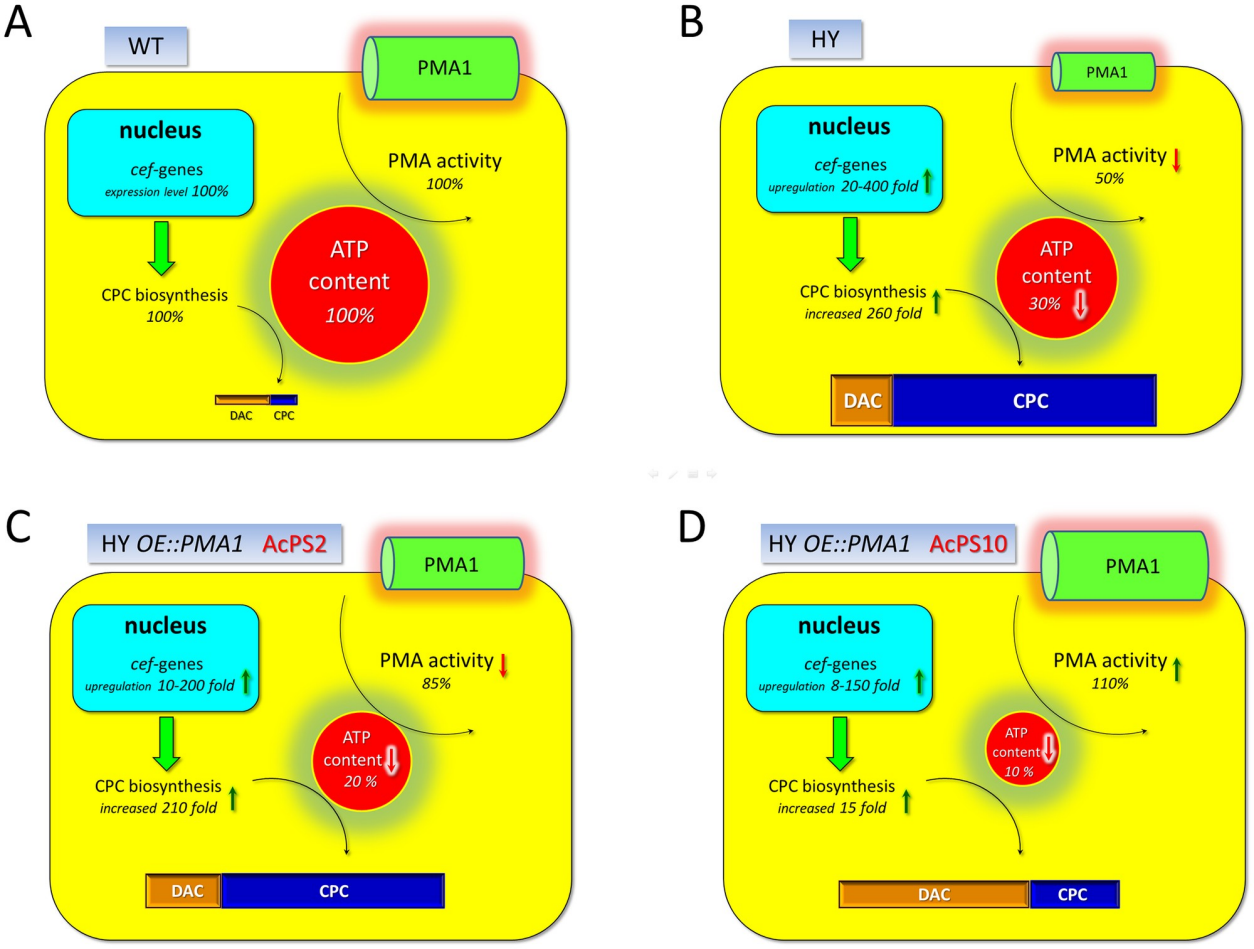
The ATP consumption by PMA activity and CPC production in *A*. *chrysogenum*. 120 h of fermentation of *A*. *chrysogenum* strains: WT (A), HY (B), AcPS2 (C) and AcPS10 (D). DAC–deacetylcephalosporin C, CPC–cephalosporin C.

In WT strain, the PMA activity is about 5,5 nmol Pi/min/mg total cell protein, ATP content is ~3,5 μmol/ g dry biomass, CPC production is ~35 μg/ ml (and DAC production is 50–100 μg/ ml). In HY strain PMA activity decreased to 50%, ATP content is depleted about three fold (up to 30% of WT strain), CPC production increased 260 fold and DAC/ CPC ratio is about 10–15% (Fig 10A and 10B). The upregulation of *cef* genes (20–400 fold) in HY strain [31] occurred without duplication of beta-lactam biosynthetic clusters [5]. In HY *OE*::*PMA1* strains the PMA activity shifted to 80–110%, the ATP content is depleted to 10–20%, CPC production increased 30–250 fold, *cef* genes upregulated 8–200 fold (all values are relative the levels in WT strain) (Fig 10C and 10D). In AcPS2 strain the PMA activity is decreased to 85% relative WT strain, but increased 1.7 fold relative HY strain-recipient; ATP content is depleted to 20% from WT strain ATP content and to 1.7 fold relative HY strain; the CPC production increased 210 fold to the yield in WT strain, but drop 1.2 fold to the yield in HY strain (Fig 10C). DAC/ CPC ratio was very close to such ration in HY strain. In AcPS10 strain the PMA activity is increased to 110% relative WT and 1.7 fold relative HY strain; ATP content was depleted to 10% from WT strain ATP content and to 3 fold relative HY strain; the CPC production increased 15 fold to the yield in WT strain, but drop more than 10 fold to the yield in HY strain (Fig 10D). DAC/ CPC ratio shifted to 88.5% from 13.5% for HY strain. Our results showed that introducing *PMA1*_*sc*_ gene under the control of gpdA promotor from *A*. *nidulans* into *A*. *chrysogenum* HY strain leads to the increasing of PMA activity (Fig 5A). Also there was a downward trend between an increase in PMA activity and ATP content in different HY/ *PMA1*_*sc*_ recombinants (Fig 7B).

PMA1 is the major membrane protein in fungal cells. It is known that fungal PMA1 makes up 5–10% of the total membrane protein, occupying about one-third of the surface of the cytoplasmic membrane [60,77] and is the main consumer of cell`s ATP. The consumption is about 20% in yeast cells and 20–50% cell ATP in mycelial fungi [28] with up to 38–52% ATP consumed by *N*. *crassa* PMA1 [78]. At the same time, when PMA1 is inhibited by various drugs, unused ATP can accumulate in the cell [79]. It has also been shown that in PMA1 mutants with a weakened level of H^+^-ATPase activity, the level of intracellular ATP also increases [80]. It can be assumed that increasing the PMA activity leads to depletion of the ATP content in HY strain, which initially has a reduced ATP content (Fig 5B). Also, the CPC production is an ATP consuming process (Fig 7A) and there is a relationship between a decrease in ATP content and a decrease in the yield of CPC (Fig 7C and 7D). In addition, the content of the CPC biosynthetic precursor, DAC, was determined in recombinants with different PMA activity. It was shown that at the last stage, the drop in the ATP content was critical (Fig 7E). Exhaustion of endogenous ATP levels may be just one of the factors inhibiting CPC productions in PMA1_sc_-overexpressing strains and downregulation of genes for CPC biosynthesis, transport and regulation encoded by the “early” and “late”–clusters, namely *pcbAB*, *pcbC*, *cefD1*, *cefEF*, *cefG*, *cefP*, *cefM*, and *cefR*.

Another possible observation for the inhibition of CPC biosynthesis by excessive PMA activity and in particular, accumulation of DAC, may be also be explained in part by indirect effects of reduced ATP pools on cytosolic acetyl-CoA levels produced by ACS and ACL (Fig 6C). Diminished acetyl-CoA content may reduce the availability of co-substrate for CefG–the last enzyme in CPC biosynthesis pathway (Fig 7E). The increased PMA activity on gene expression may be due to alteration of intracellular pH and subsequent modulation of pH-dependent transcription of *cef* genes known to be regulated by pH-responsive PacC transcription factor [3]. PMA1 is also one of the known effectors of fungal morphology, regulating through transmembrane pH and electrical gradient the assembly of cytoskeletal components required for hyphal extension and polarized growth [70]. In this respect, it is noteworthy that all obtained transformants had typical alteration of colony morphology (Fig 3), similar to the polyamine-increased PMA1 activity during yeast to hyphae transition of *Yarrowia lipolytica* [75]. Since exogenous PAs influence the production of target SM in filamentous fungi, such as beta-lactam productions in *P*. *chrysogenum* [81] and *A*. *chrysogenum* [82], or lovastatin production in *Aspergillus terreus* [83,84], the effect of polyamines on SM biosynthesis may be also mediated through PMA1 activity.

## Conclusions

In summary, our data demonstrated the interrelationship of H^+^-ATPase activity of PMA1 and cephalosporin C (CPC) production in *A*. *chrysogenum*. In CPC high-yielding (HY) strain, the H^+^-ATPase activity is decreased, related to WT strain. The elevation of H^+^-ATPase activity in HY/ *PMA1*_*sc*_ recombinants to the level of PMA activity in WT strain leads to the downregulation of *cef* genes and decreases the CPC production by 1.2–10 fold. The reduced PMA activity in *A*. *chrysogenum* HY strain may be one of the selected events during CSI, elevating the ATP content for CPC production.

## Supporting information

S1 Table The data after MALDI–TOF and LC–MS/MS analysis of proteome from *A*. *chrysogenum* WT strain after 120 h of fermentation. * Line 255. KFH44673.1 Plasma membrane ATPase-like protein [annotated by *Acremonium chrysogenum* ATCC 11550] is filled with yellow.
(XLSX)

S2 Table The data after MALDI–TOF and LC–MS/MS analysis of proteome from *A*. *chrysogenum* HY strain after 120 h of fermentation. * Line 212. KFH44673.1 Plasma membrane ATPase-like protein [annotated by *Acremonium chrysogenum* ATCC 11550] is filled with yellow.
(XLSX)

S3 Table Pma1 activity *in situ* (nmol Pi/ min/ mg total cell protein) after incubation of fungi cells with 100 mM deoxyglucose, 15 min. * Data are means ± SD, n = 3.
(DOCX)

S1 File Materials and methods. Proteomic analysis for *A*. *chrysogenum* WT and HY strains.
(DOCX)

S1 Raw images (PDF)
