# Supplementary material for: The critical role of plasma membrane H+-ATPase activity in cephalosporin C biosynthesis of Acremonium chrysogenum
Source: PLoS One. 2020 Aug 31;15(8):e0238452. doi: 10.1371/journal.pone.0238452 (PMC7458343; doi:10.1371/journal.pone.0238452)
Supplement: S3 Table — * Data are means ± SD, n = 3. (DOCX) [file pone.0238452.s003.docx]

**S1 Table. Pma1 activity *in situ* (nmol Pi/ min/ mg total cell protein) after incubation of fungi cells with 100 mM deoxyglucose, 15 min.**

| **Strain** | **Pma1 activity^*^** | **Source** |
| --- | --- | --- |
| *S.cerevisiae* SEY 6210 | 16.8 ± 2.19 | [1] |
| *S.cerevisiae* SEY 6210/ PMA1-GFP | 5.59 ± 0.98 | [1] |
| *S.cerevisiae* SY4 | 11.6 ± 1.52 | This study |
| *S.cerevisiae* SY4/ pZEN36-Н | 12.54 ± 0.39 | This study |
| *S.cerevisiae* YPH857 | 9.31 ± 0.93 | This study |
| *S.cerevisiae* YPH857/ pZEN36b | 13.04 ± 1.42 | This study |
| *A.chrysogenum* WT | 5.5 ± 0.52 | This study |
| *A.chrysogenum* HY | 2.96 ± 0.5 | This study |
| AcPS2 | 4.75 ± 0.21 | This study |
| AcPS4 | 5.7 ± 0.33 | This study |
| AcPS6 | 5.85 ± 0.31 | This study |
| AcPS10 | 4.1 ± 0.35 | This study |
| AcPS11 | 4.3 ± 0.43 | This study |
| AcPS20 | 5.05 ± 0.24 | This study |
| AcCefT6 | 2.99 ± 0.62 | This study |

Data are means ± SD, n=3.

**References**

1. Permyakov S, Suzina N, Valiakhmetov A. Activation of H+-ATPase of the Plasma Membrane of *Saccharomyces cerevisiae* by Glucose: The Role of Sphingolipid and Lateral Enzyme Mobility. van Veen HW, editor. PLoS One. 2012;7: e30966. doi:10.1371/journal.pone.0030966
