# Supplementary material for: The critical role of plasma membrane H+-ATPase activity in cephalosporin C biosynthesis of Acremonium chrysogenum
Source: PLoS One. 2020 Aug 31;15(8):e0238452. doi: 10.1371/journal.pone.0238452 (PMC7458343; doi:10.1371/journal.pone.0238452)
Supplement: S1 File — Proteomic analysis for A. chrysogenum WT and HY strains. (DOCX) [file pone.0238452.s004.docx]

**S1 Materials and methods**

**Proteomic analysis for *A. chrysogenum* WT and HY strains**

The proteomic analysis for *A. chrysogenum* WT and HY strains after 120 h of fermentation was performed by liquid chromatography and tandem mass spectrometry

**Sample preparation**

Proteins were isolated as described earlier [1] with some modifications. Frozen fungi cells were ground to a fine powder in a precooled mortar using liquid nitrogen. 1 gram of cells was resuspended in 5 ml of 10 mm potassium phosphate buffer (pH = 7.4) containing 0.1% (w/v) DTT and 0.2% (w/v) phenylmethylsulfonyl fluoride supplemented with tablets (one tablet/10 ml of buffer) of the protease inhibitor mixture CompleteTM (Roche Applied Science). The mixture was stirred at 4°C for 2 h, and the extract was clarified twice by centrifugation at 13,300 rpm for 5 min. Proteins were precipitated for 30 min at −20°C after the addition of 1/10 volume of 100% TCA. Final pellets washed twice with cold acetone by a final wash with cold ethanol to remove traces of TCA and dried under vacuum at 45°C for 60 min. Dried proteins were reconstituted in 75 mM triethylammonia hydrocarbonate (Sigma, St. Louis, MO, USA), 6% acetonitrile and 0.25% sodium deoxycholic acid. Protein solutions were heated at 95°C for 5 min (Eppendorf, Thermomixer Comfort) to enhance denaturation. After cooling/ chilling 3 mM TCEP (tris-(2-carboxiethyl)-phosphine) (Thermo Scientific, Rockford, IL, USA) and 15 mM DTT (dithiothreitol) (Thermo Scientific, Canada) were added to reduce sulfhydryl bonds of cysteine residues. The reaction was lasted for 30 min at 40°C and continual vigorous stirring. Then 17.2 mM of 4-vinylpyridine (Sigma, St. Louis, MO, USA) solution in 30% 2-propanol (Fluka, St. Louis, MO, USA) was added for proteins alkylation. The resulting reaction mixture was incubated for 45 min at 35°C in dark place. Proteins were digested by sequencing grade modified trypsin (Promega, Madison, WI, USA). For this purpose trypsin (400 ng/µL) was added at 1:50 ratio (w/w) sequentially in two steps. The enzymatic reaction was lasted 9 h at 37°C with repeated stirring for 3 min in every 15 min to sediment condensate from tube walls. The reaction was terminated by adding formic acid (Merck, Darmstadt, Germany) to 1% at final concentration. The resulting solutions were centrifuged at 12,000 rpm for 10 min at 20°C to sediment insoluble deoxycholic acid. Supernatant was diluted up to 250 µL by distilled water (Milli-Q Intergral-3 system, 18.5 mΩ*cm2, TOC<3 ppb) and transferred into 3MWCO polyethersulfon spin filter tubes (Sartorius Stedium, Goettingem, Germany) and centrifuged for 45 min at 12,000 rpm at 25°C. The resulting filtrate was diluted by 1% formic acid up to 500 µL and loaded onto the1 cc Discovery DSC-18 (Supelco, Supelco Park, Bellefonte, PA) cartridges preconditioned with methanol (Fisher Chemical, Loughborough, UK) and 0.5% formic acid for solid-phase extraction. Peptides were eluted by 1 mL of methanol with 3% formic acid and dried under vacuum at 30°C for 30-40 min (Eppendorf Concentrator Plus, Eppendorf, Germany). The resulting dried pellet was resuspended in 20 µL of 0.5% formic acid and transferred into glass inserts with polymer feet (Agilent, Santa Clara, CA, USA) for further LC-MS analysis.

**MALDI–TOF and LC–MS/MS mass spectrometric techniques**

The peptide samples were analyzed using high resolution Q Exactive mass spectrometer (Thermo Scientific, Waltham, MA, USA). The instrument was operated in positive ionization mode and equipped with Nanospray Flex NG ion source (Thermo Scientific, Waltham, MA USA). Mass spectra were acquired in with a resolution of R=70K (normalized to m/z 400) for MS in a scan range of m/z 400 – 1200 and R=17.5K (normalized to m/z 400) for MS/MS scans. Survey MS scan was followed by selection of 20 most abundant ions for MS/MS triggering. Peptide fragmentation was performed using higher energy collisional dissociation (HCD), the collision energy was set to 27 eV (stepped ±25%) and collision gas (nitrogen) was 8 mTorr. Precursor ions were isolated within ±1 Th window and the first mass of fragmentation spectra was set to 210 m/z. Precursors were dynamically excluded from targeting for 15 s if after three sequential scans. Ions with charge states z=1+, z>5+ and ions with undefined charge states were excluded from triggering MS/MS scans. Peptides separation was performed on an Ultimate 3000 nano-flow HPLC (Thermo Scientific, Waltham, MA, USA). Prior to chromatography analysis, peptides were trapped onto enrichment PepMap C18 column (0.5 mm inner diameter, 3 mm length, 5 µm particle size) using solvent C (2% acetonitrile, 0.08% formic acid, 0.015% trifluoroacetic acid). Chromatographic separation was carried out on an analytical RSLC Acclaim PepMap C18 column (150 mm length, 75 µm inner diameter, 1.8 µm particle size, 100A pore size) using a linear gradient from 98% solvent A (water, 0.08% formic acid, 0.015% trifluoroacetic acid) and 2% solvent B (0.08% formic acid, 0.015% trifluoroacetic acid in acetonitrile) to 26% solvent B over 120 min at a flow rate of 0.3 µL/min, then increasing to 85% of solvent B for 10 minutes, washing the column in 85% of solvent B for 10 min following column equilibration at initial conditions of eluting gradient for the next 15 minutes. The total analysis time was 155 min.

Protein identification was performed using MASCOT software ([www.matrixscience.com](http://www.matrixscience.com)). All tandem mass spectra were searched against the customized proteins database originated from transcripome analysis. The following search parameters were used: Trypsin was used as the cutting enzyme, mass tolerance for the monoisotopic peptide window was set to ±10 ppm, the MS/MS tolerance window was set to ±0.02 Da and up to two missed cleavage was allowed. Cysteine pyridilethylation and oxidation of methionine were chosen as variable modifications. The criteria of positive identification were set as following: minimum score of 50, at least three positive identifications from three different runs.

**References**

1. Jami M-S, Barreiro C, García-Estrada C, Martín J-F. Proteome Analysis of the Penicillin Producer *Penicillium chrysogenum*. Mol Cell Proteomics. 2010;9: 1182–1198. doi:10.1074/mcp.M900327-MCP200
