# Supplementary material for: The critical role of plasma membrane H+-ATPase activity in cephalosporin C biosynthesis of Acremonium chrysogenum
Source: PLoS One. 2020 Aug 31;15(8):e0238452. doi: 10.1371/journal.pone.0238452 (PMC7458343; doi:10.1371/journal.pone.0238452)
Supplement: S1 Raw images — (PDF) [file pone.0238452.s005.pdf]

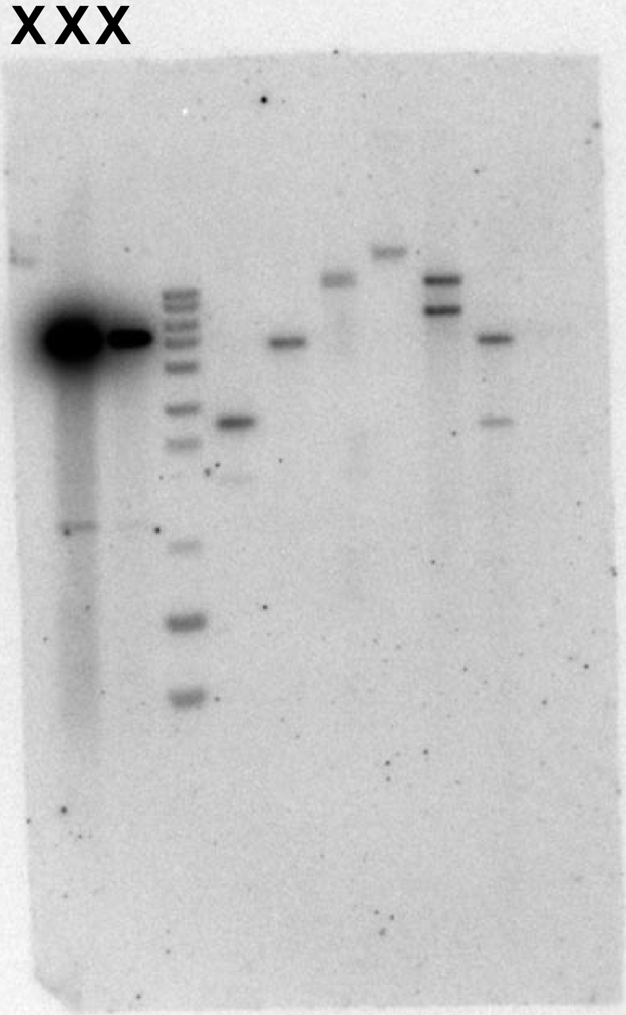
XXX

Comments to the raw image

About the S1_raw_images.pdf

Raw data file "S1_raw_images.pdf" contains one blot image that corresponds to **Fig 2A.** This is the original image from the Typhoon Trio+. Three cropped lines from the left corner are marked with an "X" above original blot image and are corresponded to: one of AcPS transformant (does not described in current manuscript) and two samples from pZEN36c plasmid with tenfold dilution (overloaded in both cases), respectively. The rest of the blot is stored completely and unchanged, as in Figure 2A.

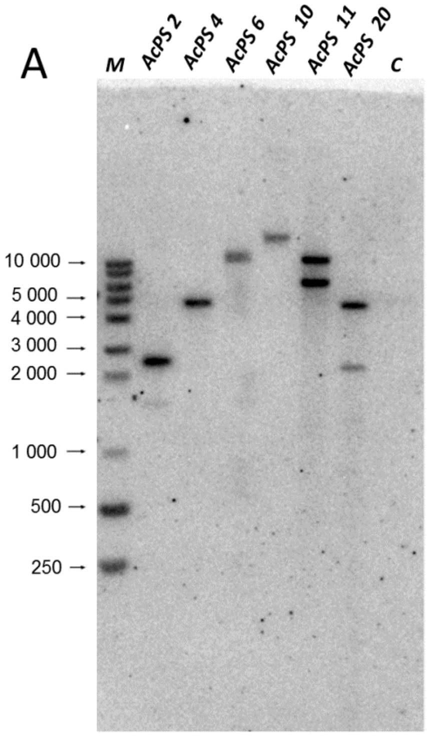

Fig 2. Analysis of *A. chrysogenum* HY/ pZEN36c transformants. (A) Southern blot hybridization of genomic DNA cleaved at *Age*I sites with a probe for screening *PMA1*$_{sc}$ sequence: *M* – GeneRuler 1 kb DNA Ladder (Thermo Fisher Scientific, USA); *AcPS 2, 4, 6, 10, 11, 20* – *Age*I-cleaved DNA of *A. chrysogenum* HY/ pZEN36c transformants; *C* – *Age*I-cleaved DNA of *A. chrysogenum* HY.

Image development

Isolated genomic DNA was treated with the *Asi*A1, separated in 1% agarose and transferred to the Amersham Hybond-XL membrane ("GE Healthcare", USA) under alkaline

transfer conditions. The DNA fragment with *PMA1*$_{sc}$*-tagYFP* sequence was obtained after PCR of pZEN36c with primers GKR1_N/ GKF1_N, labeled with DecaLabel DNA Labeling Kit ("Fermentas", Lithuania) and used in hybridization procedure. Visualization was performed using a Typhoon Trio+ phosphor imager system (“GE 164 Healthcare”, USA), with the following parameters: a 390 BP filter and a 633 nm laser. The original tif. file from Typhoon Trio+ was used to create “S1_raw_images.pdf” and is in attachment – S1_raw_images.tif
